# Multi-Biofunctional Properties of Phytofabricated Selenium Nanoparticles From *Carica papaya* Fruit Extract: Antioxidant, Antimicrobial, Antimycotoxin, Anticancer, and Biocompatibility

**DOI:** 10.3389/fmicb.2021.769891

**Published:** 2022-02-17

**Authors:** Swetha Reddy Vundela, Naveen Kumar Kalagatur, Anusuya Nagaraj, Krishna Kadirvelu, Siddaiah Chandranayaka, Kasturi Kondapalli, Abeer Hashem, Elsayed Fathi Abd_Allah, Sudhakar Poda

**Affiliations:** ^1^Department of Biotechnology, Acharya Nagarjuna University, Guntur, India; ^2^DRDO-BU Centre for Life Sciences, Coimbatore, India; ^3^Department of Biochemistry, Bharathiar University, Coimbatore, India; ^4^Department of Biotechnology, University of Mysore, Mysuru, India; ^5^Department of Botany and Microbiology, College of Science, King Saud University, Riyadh, Saudi Arabia; ^6^Department of Plant Production, College of Food and Agricultural Sciences, King Saud University, Riyadh, Saudi Arabia

**Keywords:** selenium nanoparticles, *Carica papaya*, antimicrobial activity, antimycotoxin activity, ochratoxin A, *Aspergillus ochraceus*, *Penicillium verrucosum*, *Danio rerio*

## Abstract

The present study focused on phytofabrication of selenium nanoparticles (SeNPs) from *Carica papaya* extract and exploration of their multi-biofunctional features. Total phenolics and flavonoids of *C. papaya* fruit extract were determined as 23.30 ± 1.88 mg gallic acid equivalents and 19.21 ± 0.44 mg quercetin equivalents per gram, respectively, which suggested that *C. papaya* fruit extract could be a competitive reducing and stabilizing agent during phytofabrication of nanoparticles. UV–Vis and FTIR spectroscopy showed the formation of SeNPs from sodium selenite, which could be related to the reducing and stabilizing activities of *C. papaya* fruit extract. The SeNPs were found to be stable with a Zeta potential of −32 mV. The average hydrodynamic size of SeNPs was found as 159 nm by dynamic light scattering. The SeNPs showed a broader XRD pattern with no sharp Bragg’s peaks and found to be amorphous. SEM showed that SeNPs were spherical in shape and EDX pattern showed that SeNPs were made up of Se (71.81%), C (11.41%), and O (14.88%). The HR-TEM picture showed that SeNPs were spherical in morphology and have a size range of 101–137 nm. The SeNPs exhibited potent antioxidant activity and their EC_50_ values (effective concentration required to inhibit 50% of radicals) were 45.65 ± 2.01 and 43.06 ± 3.80 μg/ml in DPPH and ABTS assays, respectively. The antimicrobial action of SeNPs was found as a broad spectrum and suppressed microbial pathogens in ascending order: fungi > Gram-positive bacteria > Gram-negative bacteria. The SeNPs have been demonstrated to reduce the growth and ochratoxin A (OTA) of mycotoxigenic *Aspergillus ochraceus* and *Penicillium verrucosum* at 40 μg/ml in broth culture, which is noteworthy. The SeNPs reduced cancer cell proliferation (RAW 264.7, Caco-2, MCF-7, and IMR-32) more preferentially than normal cells (Vero), found to be highly biocompatible. Lower doses of SeNPs (up to 50 μg/ml) were shown to be less toxic and did not cause death in *Danio rerio* (zebrafish) embryos, implying that lower doses of SeNPs could be beneficial for biological purposes. The present study concluded that phytofabricated SeNPs have multiple biofunctional properties, including antioxidant, antimicrobial, antimycotoxin, and anticancer activities, as well as high biocompatibility.

## Introduction

Nanotechnology is a cutting-edge field of research that involves regulating shape and size at the nanoscale (1–100 nm). Nanotechnology is forecast to have a transformative effect on medicine. Nanoparticles have the potential to revolutionize medicine, particularly in the diagnosis and treatment of cancer, heart diseases, and contagious diseases. Nanotechnology is poised to make a significant effect in the fields of pharmaceuticals, medical diagnostics, and agriculture (Ijaz et al., 2017; Kalagatur et al., 2018b; Khan et al., 2018a, c, 2020a, 2020b, 2021a, 2021b, 2021c; Siddaiah et al., 2018; Hassan et al., 2019; Alsharif et al., 2020; Fouda et al., 2020, 2021a,2021b,2021c; Khan and Lee, 2020; Rajan et al., 2020; Lashin et al., 2021; Salem et al., 2021; Shaheen et al., 2021; Sher et al., 2021; Soliman et al., 2021).

Among reported nanoparticles, researchers have recently shown an interest in selenium nanoparticles (SeNPs) for a variety of biological applications. Selenium is a vital component of human well-being and is found in selenocysteine, an amino acid that has been utilized to make different selenoproteins (Rayman, 2012). Although selenium has various health benefits, it also has a small therapeutic effect, which means that excessive ingestion of inorganic and organic selenium-based compounds can cause toxic effects (Reid et al., 2004). SeNPs are less harmful than both inorganic and organic selenium (Forootanfar et al., 2014; Gunti et al., 2019). Therefore, the scientific community is much attracted to explore the bio-prospectus of SeNPs. Bio-prospectus of SeNPs owns some beneficial features such as being antioxidant, antimicrobial, anticancer, anti-inflammatory, therapeutic, theranostic, anti-diabetic, and so on (Wadhwani et al., 2016; Khandel et al., 2018).

Bio-prospectus of SeNPs relies on various physicochemical properties, including shape, size, atomic geometry, structure, and stability. As a result, selecting an appropriate synthesis technique among a variety of techniques is important to achieve the desired result. Bio-prospectus of SeNPs might be achieved by using the optimal synthesis technique, which includes factors like pH, temperature, precursor dosage, and reaction time (Ramamurthy et al., 2013; Forootanfar et al., 2014; Khandel et al., 2018; Gunti et al., 2019).

To date, different chemical, biological, and physical procedures have been reported for the synthesis of SeNPs. Microwave radiation is an exceptionally productive technique in physical synthesis, and this technology uses strong electric currents to induce chemical interactions among the precursors (Panahi-Kalamuei et al., 2014). In chemical synthesis approaches, selenium salt is reduced using diverse reducing agents, for example, surfactants and acids, which end in the formation of stable SeNPs (Dwivedi et al., 2011). The chemical approach is well suited to the production of SeNPs of various physico-chemical features. Nonetheless, some chemical approaches were shown to be environmentally risky (Selmani et al., 2020). Moreover, physical and chemical approaches are tedious and expensive compared with biological approaches (Wadhwani et al., 2016).

As a result, researchers are concentrating their efforts on developing environmentally acceptable and low-cost methods for synthesizing SeNPs. In this regard, synthesis of SeNPs utilizing biological strategies is a unique process that is both environmentally friendly and cost-effective (Wadhwani et al., 2016). As a respect, the biological way of producing SeNPs is also regarded as a green or sustainable tactic. In this method, bacteria, fungus, and yeast, as well as algae and plants, serve as reducing and stabilizing mediators in the formation of SeNPs (Forootanfar et al., 2014; Chandramohan et al., 2018; Gunti et al., 2019). Metabolites from plant extracts, including proteins, carbohydrates, flavonoids, phenolics, vitamins, tannins, alkaloids, saponins, potassium, iron, calcium, and other compounds, act as excellent reducing and stabilizing mediators compared with chemical and physical techniques to meet the growing interest for green SeNPs synthesis (Gunti et al., 2019; Menon et al., 2019; Matai et al., 2020).

The focus of the research was to synthesize nanoparticles using *Carica papaya* fruit extract, which is environmentally safe, non-toxic, biocompatible, and affordable, and to investigate their biological capabilities, such as antioxidant, antimicrobial, antimycotoxin, and anticancer activities. *C. papaya* fruits include a wide range of secondary metabolites (phenolics, flavonoids, and tannins), which have been investigated as potential reducing agents for nanoparticle formation (Asghar et al., 2016). The qualities of *C. papaya* fruit are well recognized in herbal remedies, substantial progress has been made over the last few decades in terms of bioactivity as well as a therapeutic application for the treatment of various maladies, and it is today regarded as the most affordable valuable nutraceutical fruit (Krishna et al., 2008).

Based on the beneficial features of *C. papaya* and high acceptability from consumers, aqueous extract of *C. papaya* fruit was used as a reduction and stabilization agent in the conversion of sodium selenite (precursor) to SeNPs. The phytofabricated SeNPs were characterized and investigated for their bio-prospectus, including antioxidant, antimicrobial, antimycotoxin, anticancer, and biocompatibility.

The imbalance between the formation of reactive oxygen species and antioxidant defenses is known as oxidative stress. Oxidative stress can harm cells, proteins, and DNA, contributing to the aging process. Oxidative stress has a role in the development of a variety of disorders, including diabetes, cancer, and neurodegenerative ailments like Alzheimer’s (Tang et al., 2012). The antioxidant activity of SeNPs was determined using the DPPH and ABTS assays, and the antioxidant properties of SeNPs could be highly beneficial in the treatment of oxidative stress–related disorders.

From all around the world, there are alarming reports of new and emerging diseases caused by pathogens (Sundararaj et al., 2019). The number of multidrug-resistant bacteria is rapidly increasing. Microbe-caused secondary infections in AIDS and cancer patients are often deadly and difficult to treat. As a result, a new generation of antimicrobial medicines that are effective, safe, and can be employed to treat multidrug-resistant microbial diseases is urgently needed. Researchers proved that nano antimicrobials could provide efficient treatments for novel and multidrug-resistant bacteria (Ijaz et al., 2017; Khan et al., 2018a,c; Fouda et al., 2020). In the present study, antimicrobial action of SeNPs was tested on a wide spectrum of pathogens by micro-well dilution technique.

Mycotoxins are common contaminants of foodstuffs (Aiyaz et al., 2020). Every year, fungus and their mycotoxins contaminate around a quarter of the food sources worldwide (Alshannaq and Yu, 2017). Mycotoxins have a wide spectrum of negative health impacts, from acute toxicity to long-term consequences like immunological dysfunction and cancer (Maroli et al., 2019). The possible role of SeNPs as antifungal and antimycotoxin agents was investigated by broth culture technique.

Cancer is the cause of the majority of death rates worldwide, with approximately 10 million fatalities estimated by 2020 (Ferlay et al., 2020). Nowadays, several therapeutics are available to treat cancer; however, they take too long to cure cancer and can even have significant side effects in patients. Therefore, scientists are constantly investigating novel cancer therapeutics (Falzone et al., 2018). In the present study, phytofabricated SeNPs were investigated for anticancer activity and their selective growth suppression toward cancer cells was revealed compared with normal cells. The anticancer mechanism of SeNPs was investigated by measuring lactate dehydrogenase (LDH), mitochondrial membrane potential (MMP), and caspase-3 levels. In addition to the hunt for novel nanotechnology uses, there is a responsibility to understand the impact of these nanoparticles on living organisms and to safeguard them from possibly hazardous exposure. Therefore, in the present study, biocompatibility of SeNPs was investigated using the *in vivo* model *Danio rerio* (zebrafish) in addition to their beneficial features.

## Materials and Methods

### Chemicals and Reagents

The chemicals and reagents used in the study are provided in the Supplementary Data [(1) Chemicals and reagents].

### Collection, Preparation, and Chemical Profile of *Carica papaya* Fruit Extract

The fresh *Carica papaya* fruits were bought from the fruit marketplace, Vijayawada, India. Fruits were thoroughly washed with purified water, pulp cut into pieces by the sterilized blade, and crushed using a mixer grinder. Next, the fruit extract was sieved through a muslin cloth and Whatman no. 1 filter paper. The filtered extract was utilized for estimation of total phenolics and flavonoids, and synthesis of SeNPs.

#### Quantification of Total Phenolics

The Folin–Ciocalteu assay was used to quantify the total phenolics of *C. papaya* fruit extract (Gunti et al., 2019). The methodology is provided in the Supplementary Data [(2) Quantification of total phenolics].

#### Quantification of Total Flavonoids

Aluminum chloride colorimetric test was used to quantify the total flavonoids of *C. papaya* fruit extract (Gunti et al., 2019). The methodology is provided in the Supplementary Data [(3) Quantification of total flavonoids].

### Phytofabrication and Characterization of Selenium Nanoparticles

Briefly, 100 ml of 10 mM sodium selenite was taken in a fresh beaker of 500 ml, and to this 20 ml of fine filtrate of *C. papaya* fruit extract was added dropwise. The mixture was slowly blended at a speed of 150 rpm at room temperature for a period of 12 h and the successful phytofabrication of SeNPs was confirmed by the color change of the solution to brick red (Gunti et al., 2019). The SeNPs were subsequently separated by centrifugation for 45 min at 16,000 rpm, which was repeated 2–3 times. The SeNPs were collected and vacuum dried in an oven to obtain nanoparticles at 45°C for 12 h, which were subsequently used for characterization and bio-potential functionality. The methodology used for the characterization of SeNPs is provided in the Supplementary Data [(4) Phytofabrication and characterization of SeNPs].

### Antioxidant Activity of Selenium Nanoparticles

#### DPPH Assay

The *in vitro* DPPH radical scavenging potential of SeNPs was determined using the technique of Sellamani et al. (2016). The methodology is provided in the Supplementary Data [(5) DPPH assay].

#### ABTS Assay

Adopting the approach of Gunti et al. (2019), the *in vitro* ABTS radical scavenging activity of SeNPs was measured. The methodology is provided in the Supplementary Data [(6) ABTS assay].

### Antimicrobial Activity of Selenium Nanoparticles

The minimum inhibitory concentration (MIC) and minimum bactericidal/fungicidal concentration (MBC/MFC) of SeNPs were measured using the micro-well dilution technique as per instructions of the Clinical & Laboratory Standards Institute (Tarpay et al., 1980; Wolfensberger et al., 2013; Nagaraj and Samiappan, 2019). The antimicrobial activity of SeNPs was evaluated using different strains of Gram-positive bacteria (*Staphylococcus aureus*—MTCC 96, *S. aureus—*ATCC 13565, *S. aureus—*ATCC 14458, *S. aureus—*ATCC 19095, *Listeria monocytogenes*—MTCC 657, and *Enterococcus faecalis*—MTCC 439), Gram-negative bacteria (*Escherichia coli*—MTCC 41 and *Pseudomonas aeruginosa*—MTCC 741), and fungi (*Penicillium verrucosu*m—ITCC 2986, *Aspergillus ochraceus*—ITCC 2454, *A. oryzae*—MTCC 634, *Fusarium anthophilum*—MTCC 10129, *Rhizopus stolonifer*—MTCC 4886, *A. brasiliensis*—MTCC 1344, and *A. flavus*—MTCC 1883). The ATCC stands for the American Type Culture Collection, MTCC stands for the Microbial Type Culture Collection and Gene Bank, and ITCC stands for the Indian Type Culture Collection.

#### Antibacterial Activity

The antibacterial activity of SeNPs was studied by the micro-well dilution technique (Zapata and Ramirez-Arcos, 2015). The methodology is provided in the Supplementary Data [(7) Antibacterial activity].

#### Antifungal Activity

The antibacterial activity of SeNPs was studied by the micro-well dilution technique (Kalagatur et al., 2020). The methodology is provided in the Supplementary Data [(8) Antifungal activity].

### Antimycotoxin Activity of Selenium Nanoparticles

Selenium nanoparticles were tested for antimycotoxin action against ochratoxin A (OTA) in *Penicillium verrucosum*—ITCC 2986 and *Aspergillus ochraceus*—ITCC 2454.

#### Treatment of *S*elenium Nanoparticles

In a 250-ml Erlenmeyer flask, different doses of SeNPs (5, 10, 20, 30, and 40 μg/ml) were introduced to 100 ml of sterile Sabouraud dextrose broth. Following that, 10 μl of *P. verrucosum* and *A. ochraceus* spore suspension (10^6^ spores/ml) were distinctly inoculated and allowed to grow for 14 days at 28°C. The fungus that was not treated with SeNPs served as the control. The fungal mycelium was isolated from the growing media after 14 days of growth by filtration with Whatman no. 1 filter paper. Following that, mycelia and the obtained filtrate were analyzed for fungal growth and OTA, respectively.

#### Quantification of Fungal Growth and Ochratoxin A

The quantification of fungal growth and OTA was as per the methodology of Kalagatur et al. (2020). The methodology is provided in the Supplementary Data [(9) Quantification of fungal growth and OTA].

### Anticancer and Biocompatibility of Selenium Nanoparticles

Anticancer activity of SeNPs was assessed on murine macrophage cells (RAW 264.7), human colorectal adenocarcinoma (Caco-2), human mammary gland adenocarcinoma (MCF-7), and human neuroblastoma (IMR-32) cells. The biocompatibility of SeNPs was assessed on normal cell line Vero (kidney epithelial cells from an African green monkey). The cells were acknowledged from the repository cell line center of the National Centre for Cell Science (NCCS), Maharashtra, India. The cells were grown in DMEM media with 10% fetal bovine serum (FBS) in a humidified incubator at 37°C, 5% CO_2_, and 95% air. The cells were cultured in 75-cm^2^ flasks with alternate days of medium replenish and confluent cells of 85–90% were used in the experiment.

The stock of SeNPs was prepared in DMSO (10 mg/ml) and the experimental test concentration of SeNPs (up to 120 μg/ml) was then prepared in DMEM without FBS. The concentration of DMSO was kept constant throughout the investigation at 0.01%. The cells treated alone with DMEM containing 0.01% DMSO served as a control in anticancer and biocompatibility studies. Cisplatin was used as a standard anticancer agent (positive control). The stock of cisplatin was made in DMSO (10 mg/ml), and test concentrations of cisplatin (up to 10 μg/ml) were made in DMEM without FBS. The concentration of DMSO in the cisplatin test samples was kept at 0.01%.

#### Cell Viability and Micro-Morphology Assessment

The cytotoxic effects of SeNPs on both normal and cancerous cell populations were measured using the MTT assay, which was determined by the cellular proliferation rate and cell viability (Swaminathan et al., 2019, 2021; Kota et al., 2021). The methodology is provided in the Supplementary Data [(10) Cell viability and micro-morphology assessment].

#### Anticancer Mechanism

The anticancer mechanism of SeNPs on RAW 264.7, Caco-2, MCF-7, and IMR-32 was assessed by measuring LDH, MMP, and caspase-3 levels.

The cell viability was quantified as the amount of LDH released, which relies on plasma membrane impairment (Kalagatur et al., 2021). The methodology is provided in the Supplementary Data [(11) LDH analysis].

The MMP is a crucial aspect in the synthesis of ATP molecules and a measure for mitochondrial function and assay was performed as per the technique of Kalagatur et al. (2018a) and Maroli et al. (2019). The methodology is provided in the Supplementary Data [(12) MMP analysis].

Caspase-3 belongs to the CED-3 subfamily and is involved in the processing of procaspases 2, 6, 7, and 9, which are key regulatory enzymes in the apoptosis process. The effect of SeNPs on caspase-3 levels was measured using a caspase-3 assay kit according to the technique of Kalagatur et al. (2018a) and manufacturer’s instructions (Sigma-Aldrich). The methodology is provided in the Supplementary Data [(13) MMP analysis].

### *In vivo* Toxicological Assessment in *Danio rerio*

The experiment contemplated investigating the toxicity of SeNPs in the embryogenesis of *D. rerio* (zebrafish) as per guidelines of OECD (2013). The methodology is provided in the Supplementary Data [(14) *In vivo* toxicological assessment in *Danio rerio*].

### Statistical Analysis

The experiments in the study were carried out three times, each time independently. The mean and SD of the triplicate research were calculated using the results from each experiment. One-way ANOVA was used to analyze the statistical data, and Tukey’s test was used to determine significance among the test samples. Significant was defined as a *p*-value ≤ 0.05, and non-significant was defined as a *p*-value greater than 0.05.

## Results and Discussion

### Chemical Profile of *Carica papaya* Fruit Extract

**FIGURE 1:**
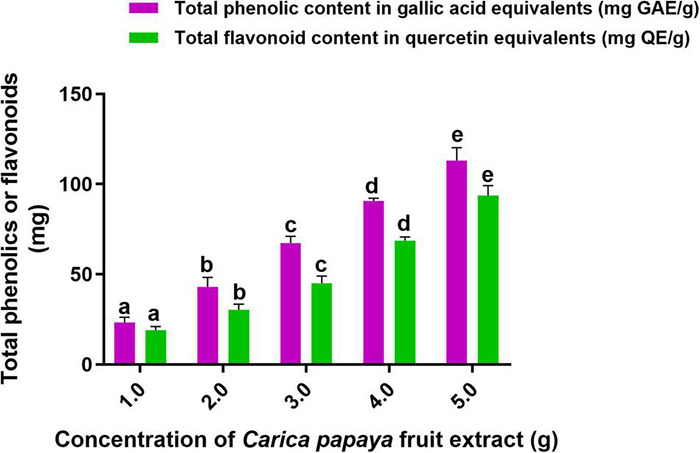
Total content of phenolics and flavonoids in *C. papaya* fruit extract. The experiments in the study were carried out three times, each time independently. The mean and SD of the triplicate research were calculated using the results from each experiment. The bar graphs with different letters within the particular analysis were significant (*p*-value ≤ 0.05) by Tukey’s test.

In this investigation, an aqueous fruit extract of *C. papaya* was prepared, and its competence in the phytofabrication of SeNPs was assessed by estimating the secondary metabolites, i.e., total phenolics and flavonoids. The total phenolic and flavonoid content of *C. papaya* fruit extract was determined using the Folin–Ciocalteu and aluminum chloride techniques, respectively. Total phenolics and flavonoids were measured in *C. papaya* fruit extracts weighing 1, 2, 3, 4, and 5 g (Figure 1). Total phenolics and flavonoids were found to be 23.30 ± 1.88 mg GAE and 19.21 ± 0.44 mg QE per gram of *C. papaya* fruit extract, respectively. At 2, 3, 4, and 5 g of *C. papaya* fruit extract, the amount of total phenolics and flavonoids was determined in ascending order. According to our findings, *C. papaya* fruit extract was found to be a potential source of phenolics and flavonoids, which are consistent with previously published documents. Asghar et al. (2016) reported total phenolics and flavonoids per gram of *C. papaya* aqueous fruit extract were 37.78 ± 0.11 mg GAE and 12.06 ± 0.20 mg catechin equivalents, respectively.

The phenolics and flavonoids of plants play an effective role in nanoparticle synthesis as reducing and stabilizing agents. The plant-based synthesis process of nanoparticles has been proven scalable, environmentally safe, and appropriate with the usage of the material for biological purposes. The foremost advantage of plant-based synthesis strategies over traditional chemical and physical methods is that they are more environmentally friendly, less expensive, and at ease to expand for vast nanoparticles synthesis. They also do not necessitate high pressures and temperatures, hazardous materials, and sophisticated equipment (Khandel et al., 2018). In compliance, the study concluded that phenolic- and flavonoid-rich *C. papaya* fruit extract could be highly applicable for the phytofabrication of SeNPs.

### Synthesis and Characterization of Selenium Nanoparticles

**FIGURE 2:**
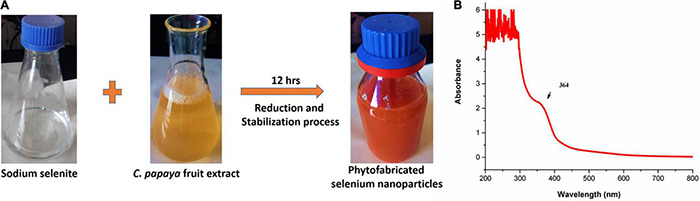
**(A)** Biosynthesis of SeNPs from *C. papaya* fruit extract. **(B)** UV–Vis spectroscopy of SeNPs.

In an aqueous solution, precursor sodium selenite is a colorless inorganic analog of the trace element selenium. Sodium selenite transforms from a colorless to a brick-red color product during the reduction reaction process, indicating the formation of nanoparticles (Gunti et al., 2019). In our study, the color of sodium selenite solution changed from colorless to ruby red over 12 h, indicating that SeNPs were biosynthesized from sodium selenite by reduction and stabilizing activity of *C. papaya* fruit extract (Figure 2A). The color shift could be caused by surface plasma resonance (SPR) (Ramamurthy et al., 2013).

The primary confirmation of SeNP biosynthesis was established by UV–visible spectroscopy (200–400 nm) based on the principle of SPR. The SPR is defined as a resonance effect caused by the interaction of metal nanoparticles’ conduction electrons with incoming photons (Gonzalo et al., 2003). In our study, single absorption peaks have been noticed at 364 nm (Figure 2B). The single peak represents typical spherical morphology nanoparticles, and it can be attributed to the fact that a single SPR band reflects spherical particles, while more than one SPR band denotes anisotropic compounds (Jain et al., 2015). In previous studies, researchers have found that phytofabricated SeNPs exhibit UV–visible maximum absorption in different ranges. According to our results, Menon et al. (2019) noticed a unique peak for ginger-mediated phytofabricated SeNPs between 370 and 420 nm and determined that the SeNPs were spherical. Similarly, Ramamurthy et al. (2013) synthesized SeNPs from fenugreek extract and found a peak between 200 and 400 nm, with 390 nm being the highest absorbance peak. Also, Alagesan and Venugopal (2019) produced spherical SeNPs from *Withania somnifera* and observed a maximum absorbance of 310 nm. The preliminary findings of UV–visible spectroscopy confirmed that *C. papaya* fruit extract has successfully reduced and stabilized the sodium selenite to SeNPs.

**FIGURE 3:**
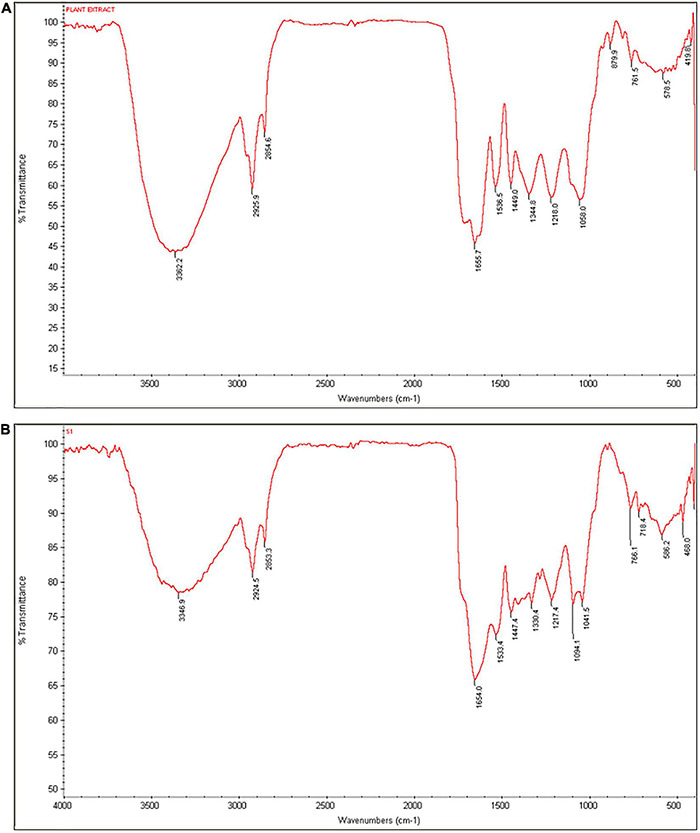
FTIR spectra of **(A)** *C. papaya* fruit extract and **(B)** SeNPs.

Next, reduction and stabilization activity of *C. papaya* fruit extract in the biosynthesis of SeNPs was investigated using FTIR spectroscopy. The FTIR technique reveals the functional groups that exist on the surface of nanoparticles by measuring chemical bond vibrational rates (Khan et al., 2018b). Figures 3A,B shows the comprehensive spectra of *C. papaya* fruit extract and biosynthesized SeNPs, respectively. Multiple prominent bands in the FTIR spectrum of *C. papaya* fruit extract correspond to the functional groups of phenolics and flavonoids. In FTIR spectrum of *C. papaya* fruit extract, wavenumbers at 3362, 2925, 2854, 1655, 1536, 1449, 1344, 1218, 1058, 879, and 761 cm^–1^ represented for H-bonded stretching in O–H group, C–H asymmetrical stretch vibration of alkanes, carboxylic acid O–H stretch, N–H bending vibrations of amide I bond, NH_2_ deformation (amide II), C = C aromatic, CH_3_ C–H bending in alkyls, R–O–R (ether), C–O stretch, C–C stretching vibration, and aromatic C–H out-of-plane bending absorptions, respectively. In the process of SeNP synthesis (reduction and stabilization), the band at 3,362 cm^–1^ of *C. papaya* fruit extract that corresponds to the O–H group has been relocated to 3,346 cm^–1^ in SeNPs, suggesting that hydroxyl groups played a major role in the bonding of SeNPs during the synthesis process. Similarly, 1,655 cm^–1^ (N–H bending vibrations of the amide I bond) and 1,536 cm^–1^ (NH_2_ deformation of amide II) of *C. papaya* fruit extract were shifted in SeNPs and confirmed that amide groups were engaged in SeNP synthesis. In addition, a significant shift was seen at 1,058 cm^–1^ (C–O stretch) in *C. papaya*, which was found to be shifted to 1041 cm^–1^ in SeNPs, confirming the function of C–O stretch in SeNP synthesis. According to the findings of the FTIR analysis, *C. papaya* fruit extract was successful in reducing and stabilizing the sodium selenite to SeNPs.

**FIGURE 4:**
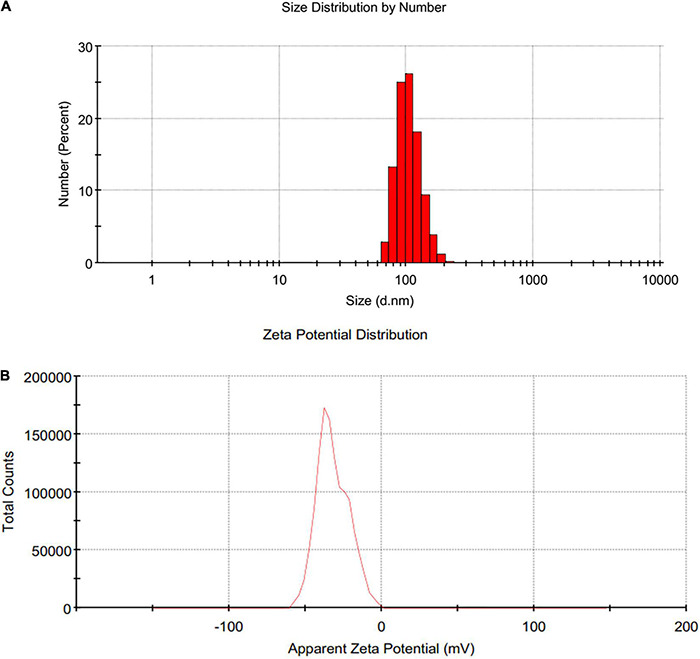
**(A)** DLS and **(B)** Zeta potential of SeNPs.

Dynamic light scattering (DLS) pattern and Zeta potential have been used to determine the hydrodynamic size and effective electric charge on the surface of SeNPs, respectively. DLS technique is used for determining the hydrodynamic size distribution profile of nanoparticles suspended in the liquid. DLS uses random changes in the intensity of light scattered from a suspension of nanoparticles to measure nanoparticle size. The DLS pattern in our investigation revealed that the synthesized SeNPs were nanosized, with an average dynamic size of 159 nm (Figure 4A). The lack of multiple peaks in the DLS pattern indicated that SeNPs were monodispersed. The polydispersity index for SeNPs was determined to be < 0.2, indicating that SeNPs were found to be in a less aggregated state.

The sustainability of a nanomaterial is driven by its Zeta potential, which is critical for the material’s existence and function (Clogston and Patri, 2011). Because of the larger electrical repulsion between them, the material with a higher magnitude of Zeta potential tends to be more stable. The Zeta potential for SeNPs was found to be −32 mV in this investigation, indicating that SeNPs were negatively charged (Figure 4B). Thus, presence of negative electro-static forces favors being SeNPs in dispersion form. The phenolics and flavonoids in *C. papaya* fruit extract may be responsible for the negative charge provided to SeNPs. In accordance with our findings, Kokila et al. (2017) and Gunti et al. (2019) showed that SeNPs produced from *Diospyros montana* leaf extract and *E. officinalis* fruit extract had negative charges as well.

**FIGURE 5:**
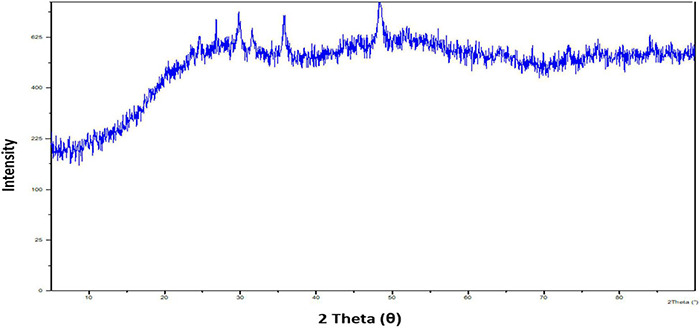
X-ray diffraction pattern of SeNPs.

Following, X-ray diffraction (XRD) examination confirmed the nature of the synthesized SeNPs (Figure 5). The XRD pattern has indicated broader peaks and deprived of any sharp Bragg’s peaks, which shows that SeNPs were amorphous in nature. According to our observations, Li et al. (2007) and Gunti et al. (2019) synthesized SeNPs from *Capsicum annuum* leaf extract and *Emblica officinalis* fruit extract, respectively, and described them as amorphous and shapeless. The irregular and arrayed selenium atoms in the form of disorganized chains may be responsible for SeNPs’ amorphous nature (Van Overschelde et al., 2013; Gunti et al., 2019).

**FIGURE 6:**
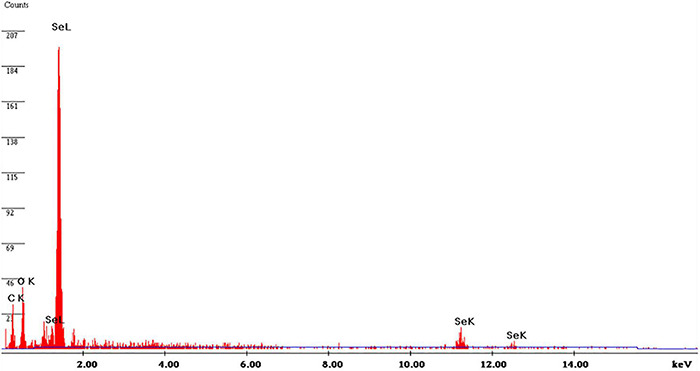
Energy-dispersive X-ray pattern of SeNPs.

Energy-dispersive X-ray (EDX) analysis was used to assess the elemental chemical components of SeNPs. Figure 6 shows the EDX pattern of SeNPs, which revealed that SeNPs include Se (71.81%), C (11.41%), and O (14.88%). The SeNPs were found to be mostly made up of selenium. In addition, SeNPs were found to include lower quantities of C and O, which could be attributable to phenolics and flavonoids of *C. papaya* fruit extract. In support of our study, Gunti et al. (2019) synthesized SeNPs from *E. officinalis* fruit extract and reported that SeNPs are chemically composed up of Se, C, and O, with Se being the main constituent. Similarly, Matai et al. (2020) phytofabricated SeNPs from *Phyllanthus emblica* and showed that SeNPs are primarily made up of Se.

**FIGURE 7:**
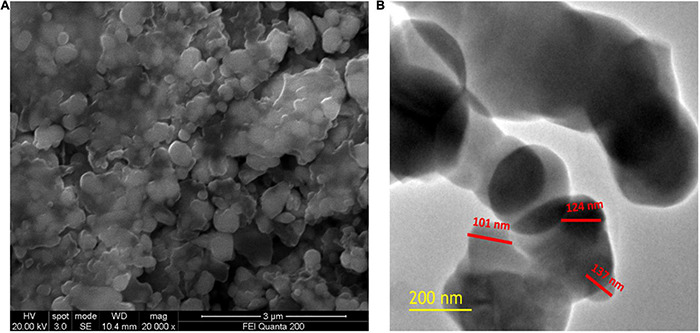
**(A)** SEM image of SeNPs. **(B)** HR-TEM image of SeNPs.

Scanning electron microscopic (SEM) analysis was used to assess the micromorphology of SeNPs. Figure 7A depicts the SEM observation of SeNPs. The SeNPs were observed in a modest aggregation form with a spherical shape. The findings were comparable with those of Kokila et al. (2017), Gunti et al. (2019), and Matai et al. (2020), who found spherical SeNP synthesis mediated by plant extracts. High-resolution transmission electron microscopy (HR-TEM) analysis was used to confirm the form and size of SeNPs as shown in Figure 7B. The HR-TEM results of SeNPs were consistent with the DLS and SEM results. The shape of SeNPs was spherical and found to be in the size range of 101–137 nm. The increased size of SeNPs was noticed in the DLS pattern when compared with the TEM pattern, which could be owing to the presence of water molecules and *C. papaya* fruit extract coating on the surface of SeNPs (Matai et al., 2020). In support with present findings, investigators phytofabricated SeNPs from plant extracts of *Diospyros montana* leaf (Li et al. (2007), *Capsicum annuum* (Ramamurthy et al., 2013), and *Allium sativum* (Anu et al., 2017) and determined SeNPs size like 80, 50–150, and 40–100 nm, respectively.

### Antioxidant Activity of Selenium Nanoparticles

Antioxidants are molecules that prevent or delay the damage caused by free radicals to the biological system. Free radicals are highly unstable molecules produced by the physiological system in response to stress factors such as contaminants, pathogens, toxins, radioactive substances, and other forms of stress. Free radicals are reactive oxygen species (ROS) that cause oxidative stress–induced immune dysfunction, leukemia, rheumatism, Parkinson’s, emphysema, heart attacks, respiratory ailments, and other metabolic disorders (Hassan et al., 2019). Consequently, scavenging free radical molecules is critical for maintaining the vitality of a biological process. Antioxidants are hydrogen and electron donors, peroxide decomposers, singlet oxygen quenchers, radical scavengers, enzyme inhibitors, synergists, and metal-chelating agents. Antioxidant molecules are referred to as “free-radical scavengers.” Antioxidants can come from both natural and synthetic sources. Natural antioxidants include flavonoids, phenolics, tannins, and phytoestrogens (Fang et al., 2002).

In the present study, *C. papaya* fruit extract was found to contain a good amount of phenolics and flavonoids. Earlier studies have shown that *C. papaya* fruit extract possesses potential antioxidant activity (Krishna et al., 2008; Asghar et al., 2016). In addition, selenium (Se) is regarded as a strong antioxidant (Rayman, 2012). Thus, phytofabricated SeNPs could contain antioxidant capabilities of *C. papaya* fruit extract and Se; thereby, SeNPs possibly have robust antioxidant activity that could find an appropriate role in the bio-prospectus. In this work, antioxidant activity of SeNPs was measured using DPPH and ABTS scavenging tests.

**FIGURE 8:**
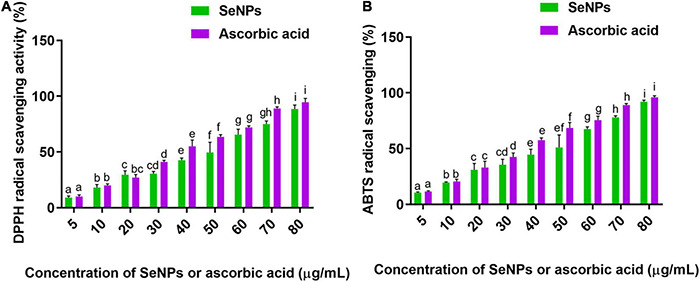
**(A)** DPPH and **(B)** ABTS radicals scavenging potential of SeNPs in a dose-dependent manner. The experiments in the study were carried out three times, each time independently. The mean and SD of the triplicate research were calculated using the results from each experiment. The bar graphs with different letters within the particular analysis were significant (*p*-value ≤ 0.05) by Tukey’s test.

The antioxidant ability of SeNPs and ascorbic acid (standard antioxidant) was tested dose-dependently in the DPPH and ABTS scavenging experiments. Both SeNPs and ascorbic acid reduced the quantity of DPPH and ABTS radicals in a dose-dependent manner, demonstrating the dose-dependent nature of antioxidant action (Figures 8A,B). SeNPs’ antioxidant activity was shown to be comparable with that of ascorbic acid, a common antioxidant. In the DPPH experiment, the EC_50_ values (effective concentration required to inhibit 50% of radicals) of SeNPs and ascorbic acid were determined to be 45.65 ± 2.01 and 38.46 ± 2.79 μg/ml, respectively. The EC_50_ values of SeNPs and ascorbic acid in the ABTS assay were determined as 43.06 ± 3.80 and 36.39 ± 2.47 μg/ml, respectively. The antioxidant potential of SeNPs was found to be comparable with that of standard antioxidant ascorbic acid, and the results were considered satisfactory. In previous studies, the antioxidant activity of SeNPs was determined to be consistent by ABTS and DPPH assays. DPPH is a well-known free radical scavenging chemical with nitrogen radical at its core and a dark purple color. SeNPs interact with DPPH by exchanging either a hydrogen atom or electrons and neutralize the DPPH free radicals. The ABTS^⋅+^ generated from the reaction between ABTS and potassium persulfate is a chemically stable blue-green chromophore compound. SeNPs convert the ABTS^⋅+^ (blue green) to ABTS (colorless) by transferring the electrons (Dumore and Mukhopadhyay, 2020). Furthermore, previous investigations by Kokila et al. (2017), Qiu et al. (2018), and Gunti et al. (2019) have revealed the antioxidant potential of SeNPs in DPPH radical scavenging assay (EC_50_) as 22.5, 500, and 15.67 ± 1.41 μg/ml, respectively.

The study determined that the antioxidant ability of SeNPs could be attributable to the selenium element and secondary metabolites of *C. papaya* fruit extract that inhabited the surface of SeNPs.

### Antimicrobial Activity of Selenium Nanoparticles

Selenium is a powerful antimicrobial agent at its core. Because of its high antifungal activity, selenium is extensively employed in the formulation of antidandruff shampoos (Van Cutsem et al., 1990). However, because of its toxicity, selenium is only used in little amounts. Recent research has focused primarily on reducing the Se toxicity and advancing the Se bio-prospectus. In this case, nanotechnology has offered a strategy for reducing toxicity and developing the Se bio-prospectus through the biosynthesis tactic (Salem and Fouda, 2021).

In the present study, the concentration of SeNPs required to inhibit (MIC) and kill (MBC/MFC) the microbial pathogens was determined by micro-well dilution assay. In the case of bacteria, MIC and MBC of SeNPs were correspondingly estimated for a period of 24 h and subsequently followed by incubation for an additional 24 h. In the case of fungi, MIC and MFC of SeNPs were determined at 24 h and subsequently followed by incubation of an additional period of 3 days, respectively. The best antimicrobial activity was noticed against fungi in relation to bacteria. The Gram-positive bacteria were relatively highly susceptible to SeNPs compared with Gram-negative bacteria. The order of antimicrobial activity of SeNPs was noticed as fungi > Gram-positive bacteria > Gram-negative bacteria. In the case of the bacteria, best MIC and MBC values of SeNPs were noticed against Gram-positive bacteria *L. monocytogenes*—MTCC 657 as 22.56 ± 3.11 and 38.56 ± 2.59 μg/ml, respectively. The least antibacterial activity of SeNPs was noticed against Gram-negative bacteria *P. aeruginosa—*MTCC 741 and its MIC and MBC were correspondingly noticed as 33.47 ± 3.09 and 61.14 ± 3.94 (Supplementary Table 1), while best antifungal activity was noticed against *A. ochraceus*—ITCC 2454 and the noticed MIC and MFC values were 16.22 ± 1.81 and 28.41 ± 4.07 μg/ml, respectively. The least antifungal activity of SeNPs was determined against *F. anthophilum*—MTCC 10129 and its MIC and MFC were determined as 27.88 ± 3.56 and 41.90 ± 5.22 μg/ml, respectively (Supplementary Table 1).

In support of our results, SeNPs have been biosynthesized from plant extract *Diospyros montana* by Kokila et al. (2017) and shown potent antimicrobial activity *A. niger*, *E. coli*, and *S. aureus*. Similarly, some other researchers have biosynthesized SeNPs from microbial sources such as *Klebsiella pneumoniae* and demonstrated potent antimicrobial activity against *Candida albicans* (Kazempour et al., 2013).

The reports concluded that biosynthesized nanoparticles produce effective antimicrobial activity in diverse ways. Particularly, potent antimicrobial activity of nanoparticles relies on the surface area that means size. The smaller magnitude of nanoparticles can readily pass across the cell wall and membrane of microbes and induce antimicrobial activity through the lysis of cells. Some in-depth antimicrobial analysis concluded that nanoparticles could cause microbial death through interfering ATP synthesis and protein functioning, and induce apoptosis pathway (Zhang et al., 2013; Salem et al., 2021).

Our study has shown the potent antimicrobial activity of SeNPs against both bacteria and fungi. Captivatingly, effective antimicrobial action was noticed against Gram-positive bacteria in relation to Gram-negative bacteria. The reason could be explained as SeNPs have robust electrostatic repugnance toward the lipopolysaccharide that is present in the membrane of Gram-negative bacteria, which has a highly negative charge. On the other hand, Gram-positive bacteria have less negative charge in relation to Gram-positive bacteria, which possibly favors the higher deposition of SeNPs on Gram-positive bacteria compared with Gram-negative bacteria (Tran et al., 2015). Therefore, SeNPs have shown potent antibacterial action against Gram-positive bacteria in comparison with Gram-negative bacteria in our investigation. Moreover, SeNPs exhibited superior antifungal potential than antibacterial potential. Instead of being an antibacterial, selenium is considered to be a powerful antifungal, and it depletes the ergosterol content and thereby induces the death of the fungi through the impairment of membranes. Until today, selenium is a major component in the formulation of anti-dandruff shampoo and is used in therapeutics of fungal infections (Pierard et al., 1997). However, the detailed antifungal mechanism of selenium is yet to be understood.

### Antimycotoxin Activity of Selenium Nanoparticles

**FIGURE 9:**
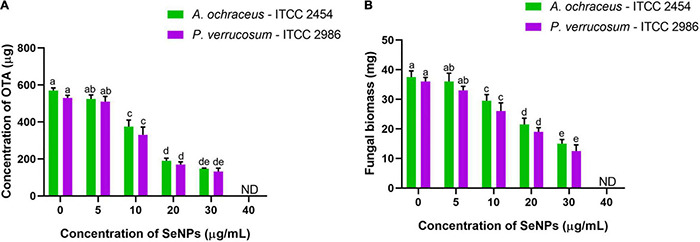
Dose-dependent inhibitory activity of SeNPs on **(A)** ochratoxin A **(B)** fungal biomass of *A. ochraceus* and *P. verrucosum*. The experiments in the study were carried out three times, each time independently. The mean and SD of the triplicate research were calculated using the results from each experiment. The bar graphs with different letters within the particular analysis were significant (*p*-value ≤ 0.05) by Tukey’s test. ND, not detected.

Antimycotoxin activity of SeNPs was tested against ochratoxigenic fungi, *A. ochrace*us—ITCC 2454 and *P. verrucosum—*ITCC 2986, at concentrations of 5, 10, 20, 30, and 40 μg/ml in broth culture for 14 days of growth period by assessing the fungal growth (biomass) and OTA content (Figure 9). Antimycotoxin activity of SeNPs was revealed by comparing with the control sample. The fungus that had not been treated with SeNPs served as the control.

In the control sample, 568.51 ± 10.88 and 527.11 ± 8.14 μg/ml of OTA were determined for *A. ochraceus*—ITCC 2454 and *P. verrucosum*—ITCC 2986, respectively, whereas fungi treated with SeNPs have shown a declined trend of OTA content. The decline of OTA content at fungal samples treated with 5 μg/ml of SeNPs has not shown a significant difference compared with control, whereas fungal samples treated with 10, 20, and 30 μg/ml of SeNPs have shown significant difference compared with control, and OTA content was not noticed at 40 μg/ml of SeNPs in both the tested ochratoxigenic fungi *A. ochraceus*—ITCC 2454 and *P. verrucosum*—ITCC 2986 (Figure 9A).

Likewise, fungal biomass of 37.5 ± 2.89 and 36.08 ± 1.22 mg was determined in the control sample of *A. ochraceus*—ITCC 2454 and *P. verrucosum*—ITCC 2986, respectively. Upon the treatment with SeNPs, fungal biomass was declined in both fungi, *A. ochraceus*—ITCC 2454 and *P. verrucosum*—ITCC 2986. A significant decline in fungal biomass was not noticed in fungal samples treated with lower concentration of 5 μg/ml of SeNPs, whereas, similar to OTA analysis, a significant decline in fungal biomass was noticed in fungal samples treated with 10, 20, and 30 μg/ml of SeNPs compared with control. Fungal growth was completely absent at 40 μg/ml of SeNPs (Figure 9B).

In the present study, SeNPs have successfully decreased the fungal biomass and OTA content in both the tested ochratoxigenic fungi, *A. ochraceus*—ITCC 2454 and *P. verrucosum*—ITCC 2986. However, fungal growth and OTA content were noticed at a lower concentration of SeNPs and could be due to the amount of SeNPs that was not adequate to decrease the fungal growth. In contrast, fungal growth and OTA content drastically declined with increasing concentration of SeNPs, and it shows the dose-dependent effect of SeNPs on fungal growth and OTA content. The OTA content was not determined at the highest concentration of 40 μg/ml of SeNPs, and it could be due to the absence of fungal growth. The fungal growth was inhibited due to the antifungal activity of SeNPs.

Only one report on SeNPs’ antimycotoxin activity is available to our knowledge. Abdel-kareem and Ahmed Zohri (2017) have demonstrated the application of SeNPs to limit the growth of *A. parasiticus*, *A. ochraceus*, and *A. nidulans*, as well as their mycotoxins aflatoxin, ochratoxin A, and sterigmatocystin, and reported high doses of SeNPs (3,000–9,000 μg/ml and 800–2,000 μg/ml, respectively) for regulating growth and mycotoxins. However, the majority of research has been done with different nanoparticles for the controlling of mycotoxins, such as zinc oxide, silver, and iron oxide (Lakshmeesha et al., 2019, 2020). However, substantial research is needed to reveal the molecular metabolic pathways that drive nanoparticle-induced antifungal and antimycotoxin action.

### Anticancer and Biocompatibility of Selenium Nanoparticles

The *in vitro* anticancer activity of SeNPs was assessed on different cancer cells such as RAW 264.7, Caco-2, MCF-7, and IMR-32 cells. The biocompatibility of SeNPs was appraised in normal cell line Vero. Cisplatin was a reference anticancer drug in the study.

**FIGURE 10:**
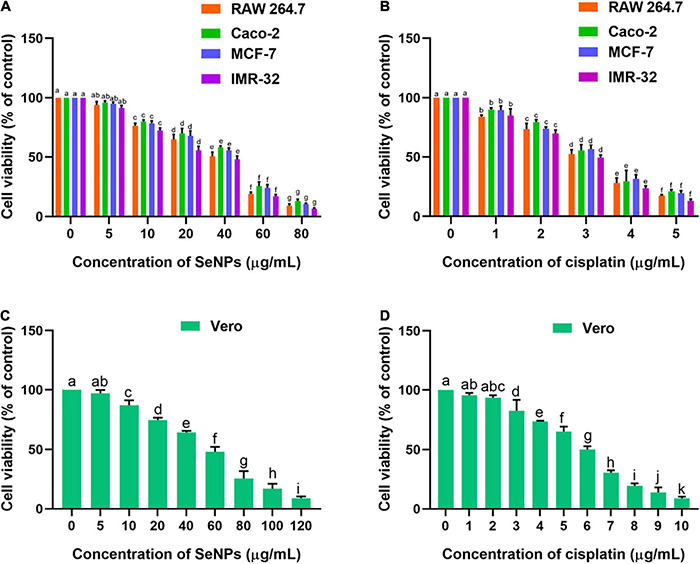
**(A)** Dose-dependent effect of SeNPs on cell viability of cancer cells determined by MTT assay. **(B)** Dose-dependent effect of cisplatin on cell viability of cancer cells determined by MTT assay. **(C)** Dose-dependent effect of SeNPs on cell viability of normal cells determined by MTT assay. **(D)** Dose-dependent effect of cisplatin on cell viability of normal cells determined by MTT assay. The experiments in the study were carried out three times, each time independently. The mean and SD of the triplicate research were calculated using the results from each experiment. The bar graphs with different letters within the particular analysis were significant (*p*-value ≤ 0.05) by Tukey’s test.

The effect of SeNPs and cisplatin on cellular proliferation rate and cell viability of normal and cancerous cell populations was measured using the MTT assay. The SeNPs and cisplatin have shown potent anticancer activity on tested cancer cells. Cisplatin has shown potent anticancer activity on all the tested cancer cells compared with SeNPs. The IC_50_ and IC_90_ values of cisplatin and SeNPs against cancer and normal cells are shown in Supplementary Table 2. The SeNPs have not shown significant anticancer activity at lower levels of 5 μg/ml when compared with control (cells not treated with SeNPs). However, a significant level of cell death (anticancer) was noticed at higher concentrations of SeNPs (10, 20, 40, 60, and 80 μg/ml) compared with the control. The superlative anticancer activity of SeNPs was noticed in brain cancer cells (IMR-32). The IC_50_ and IC_90_ values of SeNPs on IMR-32 cells were correspondingly determined as 35.98 ± 2.89 μg/ml and 71.38 ± 3.71 μg/ml. The lower anticancer activity of SeNPs was observed in Caco-2 (intestinal cancer cells). The IC_50_ and IC_90_ values of SeNPs noticed in Caco-2 cells were 42.85 ± 2.02 μg/ml and 79.55 ± 3.97 μg/ml, respectively. Overall, SeNPs and cisplatin have shown dose-dependent anticancer activity on all the tested cancer cells (Figures 10A,B). In biocompatibility analysis, SeNPs and cisplatin have been found to be highly biocompatible. The SeNPs and cisplatin have dose-dependently inhibited the cell viability of normal cells Vero (Figures 10C,D). However, SeNPs and cisplatin inhibited the cell viability of normal cells Vero at their higher concentrations in relation to cancer cells (Supplementary Table 2), which pronounced that SeNPs and cisplatin considerably inhibited the growth of cancer cells and are biocompatible toward normal cells.

The effect of SeNPs on the micro-morphology of cells was perceived by a bright-field inverted microscope (Olympus CKX53, Japan). The micro-morphological effect of SeNPs on RAW 264.7, Caco-2, MCF-7, IMR-32, and Vero cells is shown in Supplementary Figures 1–5, respectively. In the present study, cells treated with IC_50_ and IC_90_ values of SeNPs were test samples. The cells treated with the IC_50_ value of cisplatin were referred to as positive control. The cells not treated with SeNPs and cisplatin were considered as control. In cancer cells, micro-morphology of SeNP-treated cells were compared with control cells (cells not treated with SeNPs and cisplatin) and cells treated with cisplatin (positive control), and anticancer activity of SeNPs was appraised. In bright-field inverted microscopic images, control cells exhibited characteristic monolayer form and good physical shape assemblies, and reflected the well-being of the cells, whereas cells treated with IC_50_ value of cisplatin showed membrane damage and formation of membrane blebs, leakage of debris from the cells, and the formation of tiny apoptotic bodies. The cells treated with IC_50_ and IC_90_ values of SeNPs also exhibited membrane damage and formation of membrane blebs, leakage of debris from the cells, and formation of tiny apoptotic bodies. However, higher intensity of membrane damage and formation of membrane blebs, leakage of debris from the cells, and the formation of tiny apoptotic bodies were perceived at IC_90_ relative to the IC_50_ value of SeNPs. In normal cells, concentration of SeNPs required to influence the micro-morphology of normal cells was found to be much higher compared with cancer cells. The micro-morphological observation study concluded that SeNPs have selectively influenced the cellular damage in cancer cells. Thus, SeNPs were found to be biocompatible toward normal cells (Swaminathan et al., 2019, 2021).

**FIGURE 11:**
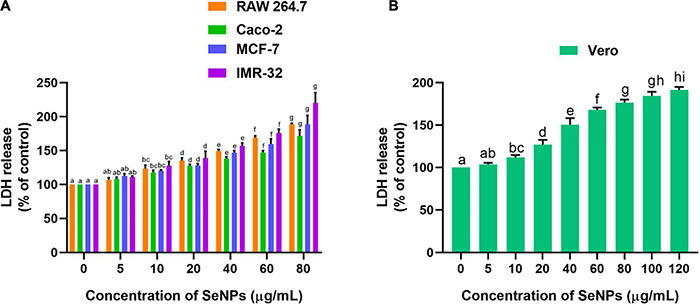
Dose-dependent effect of SeNPs on LDH leakage of **(A)** cancer cells and **(B)** normal cells. The experiments in the study were carried out three times, each time independently. The mean and SD of the triplicate research were calculated using the results from each experiment. The bar graphs with different letters within the particular analysis were significant (*p*-value ≤ 0.05) by Tukey’s test.

Following, anticancer mechanism of SeNPs in RAW 264.7, Caco-2, MCF-7, and IMR-32 cells was evaluated by LDH, MMP, and caspase-3 analysis. In our study, SeNPs have effectively influenced the viability of cancer cells through leakage of LDH, which means through impairment of cellular membrane. The low level of LDH leakage (impairment of cellular membrane) was noticed at lower concentrations of SeNPs (5 and 10 μg/ml), which was found to be not significant compared with the control. In comparison, significant LDH leakage was noticed at higher concentrations of SeNPs (20, 40, 60, and 80 μg/ml) compared with control. The obtained results in the LDH assay were found to be similar to the MTT assay. The superlative and lowest anticancer activity of SeNPs was noticed against IMR-32 and Caco-2 cells, respectively (Figure 11A). However, the concentration of SeNPs required to influence the LDH leakage in normal cells was found to be much higher compared with cancer cells (Figure 11B). The LDH study concluded that SeNPs have selectively influenced LDH leakage and membrane impairment in cancer cells. The SeNPs were found to be biocompatible toward normal cells. These results were well matched with the MTT assay. The study concluded that SeNPs have induced anticancer activity through the impairment of cellular membrane (Han et al., 2011).

**FIGURE 12:**
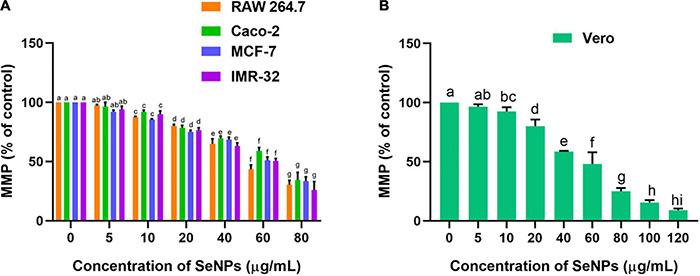
Dose-dependent effect of SeNPs on MMP of **(A)** cancer cells and **(B)** normal cells. The experiments in the study were carried out three times, each time independently. The mean and SD of the triplicate research were calculated using the results from each experiment. The bar graphs with different letters within the particular analysis were significant (*p*-value ≤ 0.05) by Tukey’s test.

The effect of SeNPs on the MMP of cancer cells was tested in a dose-dependent manner (Figure 12). We have not noticed a significant difference in MMP between the cells treated with a lower concentration of SeNPs (5 μg/ml) and control cells, while cells treated with higher concentrations of SeNPs (10, 20, 40, 60, and 80 μg/ml) have shown a significant difference in MMP compared with control cells. Much decline in MMP levels was noticed in IMR-32 cells (Figure 12A). However, in normal cell line Vero, the concentration of SeNPs required to decrease the MMP of the cells was found to be much higher compared with cancer cells (Figure 12B). The study concluded that SeNPs have selectively decreased the MMP of the cancer cells. Thus, SeNPs were found to be biocompatible toward normal cells. These results were in line with cell viability assays, i.e., MTT and LDH assays. The study concluded that SeNPs could decrease the MMP and ATP synthesis of cancer cells, and thereby promote the apoptosis of the cancer cells (Mizuno et al., 2005).

**FIGURE 13:**
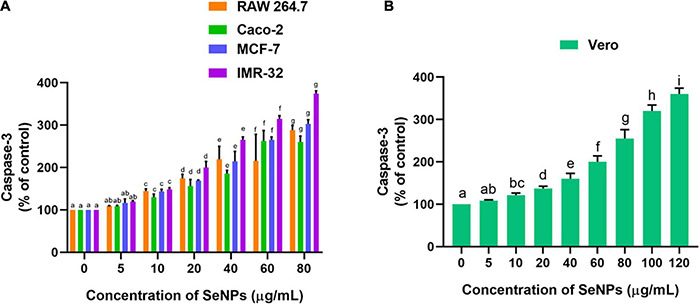
Dose-dependent effect of SeNPs on caspase-3 level of **(A)** cancer cells and **(B)** normal cells. The experiments in the study were carried out three times, each time independently. The mean and SD of the triplicate research were calculated using the results from each experiment. The bar graphs with different letters within the particular analysis were significant (*p*-value ≤ 0.05) by Tukey’s test.

The effect of SeNPs on the escalation of caspase 3 levels and apoptosis in cells was tested in a dose-dependent manner (Figure 13). Caspase-3 is known as an executioner caspase in the apoptosis process. Caspase involves a role in the devastation of cellular assemblies, for example, DNA destruction or deprivation of cytoskeletal proteins. The SeNPs have dose-dependently escalated the caspase 3 levels in cancer cells. We have noticed a significant difference in the caspase 3 levels at higher levels of SeNP-treated test samples (10, 20, 40, and 80 μg/ml) compared with control cells. The highest level of caspase 3 was noticed in IMR-32 cells and the least level of caspase 3 was noted in Caco-2 cells (Figure 13A). However, in normal cells, the concentration of SeNPs required to induce escalation of caspase 3 levels was found much higher compared with cancer cells (Figure 13B). The study concluded that SeNPs have selectively induced escalation of caspase 3 levels in the cancer cells. Thus, SeNPs were found to be biocompatible toward normal cells. The study concluded that SeNPs have induced anticancer activity through the escalation of caspase 3 levels. These results were in line with cell viability and MMP analysis.

In support of our study, earlier reports of Huang et al. (2013), Ramamurthy et al. (2013), Gao et al. (2014), and Gunti et al. (2019) determined that SeNPs enter into the cells through the mechanism of receptor-mediated endocytosis. The cancer cells exhibit an acidic pH and imbalance redox state. These conditions of cancer cells create prooxidant conversion of SeNPs and trigger the development of free radicals (Zhao et al., 2018), which causes disruption to the mitochondrial membrane and declines its potential and ceases ATP synthesis. Also, SeNPs create an endoplasmic reticulum (ER) stress. The loss of MMP, cease of ATP synthesis, and ER stress trigger apoptosis *via* activation of caspases. These conditions activate the multiple molecular pathways including the Wnt/β-catenin, MAPK/Erk, NFκB, PI3K/Akt/mTOR, and apoptotic pathways (Ramamurthy et al., 2013; Wadhwani et al., 2016; Zhao et al., 2018). However, the in-detailed safety and anticancer mechanism of SeNPs is not yet clear and needs further studies.

### Toxicity of Selenium Nanoparticles in *Danio rerio*

*Danio rerio* is in a favorable position for widespread use as an *in vivo* model vertebrate organism for toxicological assessment, owing to the in-detail of its genome project and extensive evidence obtained from developmental and genomic investigations. Moreover, size, husbandry, and early morphogenesis make *D. rerio* as an ideal toxicological model compared with other vertebrate species. In consequence, researchers frequently use *D. rerio* as an *in vivo* embryonic and larval model to study the developmental toxicity of pollutants, nanomaterials, microbial toxins, heavy metals, plastics, and other potentially toxic compounds (Hill et al., 2005).

In the present study, toxic effects of SeNPs at various doses (25, 50, 75, and 100 μg/L) on the development of *D. rerio* embryos were investigated using the fish embryo acute toxicity (FET) protocol (OECD, 2013). Embryonic developmental defects such as coagulation, non-tail detachment, lack of somite formation, opaque eyes, pericardial edema, yolk sac edema, hyperemia, absence of heartbeat, and spinal curvature were identified under an inverted microscope at various time intervals, including 24, 48, 72, and 96 hpf.

**FIGURE 14:**
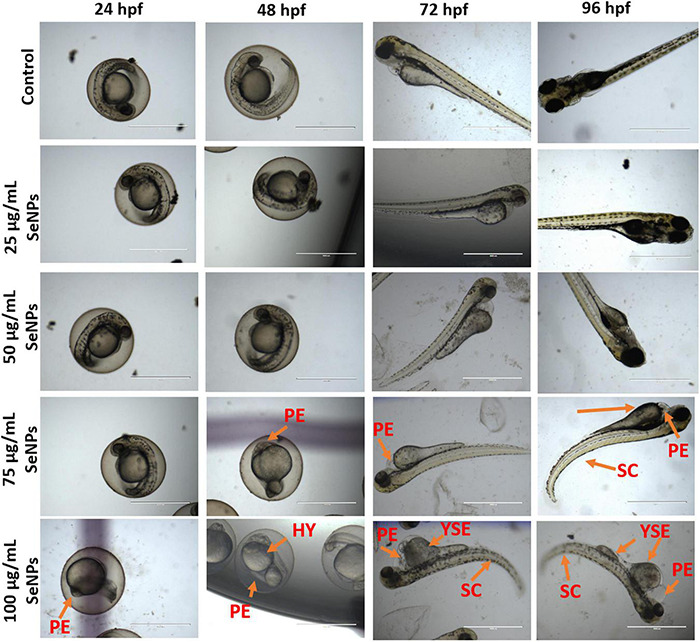
Dose-dependent toxic effect of SeNPs on embryogenesis of *D. rerio* at 24, 48, 72, and 96 hpf. PE, pericardial edema; HY, hyperemia; SC, spinal curvature; YSE, yolk sac edema. The images were captured at a magnification of ×4. Scale bar = 1,000 μm.

**FIGURE 15:**
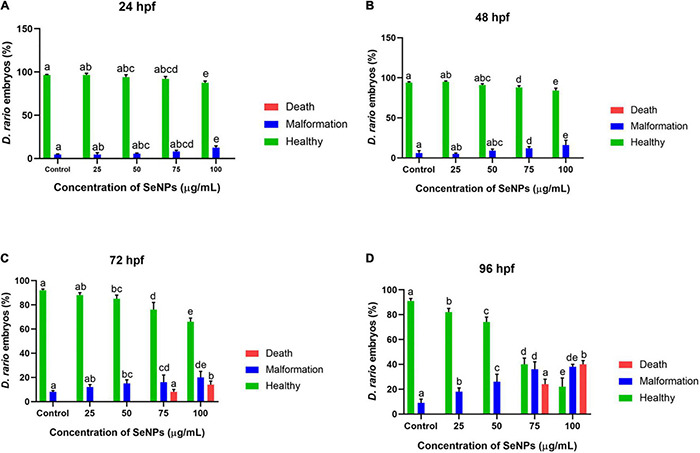
Dose-dependent effect of SeNPs on malformation and death on *D. rerio* embryos at **(A)** 24, **(B)** 48, **(C)** 72, and **(D)** 96 hpf. The bar graphs with different letters within the particular analysis were significant (*p*-value ≤ 0.05) by Tukey’s test.

The results of the study are depicted in Figures 14, 15. At 24 hpf, SeNPs had no harmful effects on embryogenesis at doses of 25, 50, and 75 μg/ml, and pericardial edema was observed at 100 μg/ml of SeNPs, but no mortality was observed at any of the tested doses of SeNPs. The SeNPs showed similar results to 24 hpf at 48 hpf, with no evidence of embryo death, and noticed hyperemia and pericardial edema at high doses of 75 and 100 μg/ml of SeNPs. At 72 hpf, SeNPs exhibited high intensity of deformities (pericardial edema, yolk sac edema, and spinal curvature) and lower death of embryos at high doses of 75 and 100 μg/ml of SeNPs. At 96 hpf, SeNPs showed toxic effects on embryogenesis and noticed embryonic death at high doses of 75 and 100 μg/ml. However, lower doses of SeNPs (25 and 50 μg/ml) did not cause embryo death at any of the time periods studied (24, 48, 72, and 96 hpf). The EC_50_ value of SeNPs was noticed as 110.18 ± 5.42 μg/ml at 96 hpf. In the present study, SeNPs were shown to be non-toxic at low levels.

Only a few reports were found to support our research. Kalishwaralal et al. (2016) reported SeNPs by one-pot green synthesis and observed the SeNPs’ toxic effects in *D. rerio* embryos at 15–25 μg/ml of SeNPs. Chandramohan et al. (2018, 2019) reported abnormalities of SeNPs on *D. rerio* embryos at beyond 10 and 30–50 μg/ml, respectively. In our study, SeNPs have not shown toxic effects and death on embryos at tested lower doses (25 and 50 μg/ml), which was found in accordance with earlier reports. The attendance of an organic layer (phenolics and flavonoids) of *C. papaya* fruit extract around SeNPs may have a significant impact on SeNPs’ toxicity.

## Conclusion

*Carica papaya* fruit extract has active ingredients such as flavonoids and phenolics, which work as potent reducing and stabilizing agents for the synthesis of SeNPs. Green synthesis mediated SeNPs have developed from *C. papaya* fruit extract in a low-cost, easier, and superior alternative to physical and chemical processes. FTIR analysis showed that SeNPs were synthesized as a result of effective interactions of functional groups of *C. papaya* fruit extract. SeNPs in aqueous solutions were found to be stable with a negative Zeta potential, which could be attributed to phytomolecules capping. The XRD pattern, SEM, and HR-TEM showed that SeNPs were amorphous in nature, spherical, and nanometer in size, respectively. The EDX pattern showed that the majority content of SeNPs was selenium. The SeNPs were found to have significant free radical scavenging ability and may be effective in the treatment of oxidative stress–related diseases. The SeNPs showed a wide range of antimicrobial activity against tested fungus, Gram-negative bacteria, and Gram-positive bacteria, indicating that SeNPs could play a role in the biomedical area as a broad-spectrum antimicrobial agent. Most fascinatingly, SeNPs are shown to decrease the growth and OTA content of mycotoxigenic *A. ochraceus* and *P. verrucosum*, implying that SeNPs could be employed in food science to prevent mycotoxin contamination. Furthermore, SeNPs preferentially inhibited cancer cell proliferation as compared with normal cells, indicating that SeNPs are highly biocompatible and could be highly recommended for usage as an anticancer drug. In addition, SeNPs were shown to be less toxic and did not cause death in *D. rerio* embryos at lower concentrations, suggesting that lower doses of SeNPs could be beneficial. The research concluded that phytofabrication of SeNPs is advantageous in a variety of biological applications, including antioxidant, antimicrobial, antimycotoxin, and anticancer, in an environmentally benign manner. According to the findings, SeNPs could be useful in medicine and the food industry. However, in-detailed toxicological trials are obligatory to establish the safety of SeNPs.

## Data Availability Statement

The original contributions presented in the study are included in the article/Supplementary Material, further inquiries can be directed to the corresponding author/s.

## Ethics Statement

Ethical review and approval was not required for the animal study because all the experiments in zebrafish were performed followed by the guidelines agreed by the Committee for the Purpose of Control and Supervision of Experiments on Animals (CPCSEA), Government of India (cpcsea.nic.in/WriteReadData/userfiles/file/SOP_CPCSEA _inner_page.pdf). Hence, this is not mandatory regarding the ethical issues, as it is not mentioned in the CPCSEA guidelines yet.

## Author Contributions

SV, NK, AN, KKa, SC, KKo, AH, EA_A, and SP contributed to conception and design of the study. SV, NK, AN, KKo, SC, AH, EA_A, and SP organized the database. SV, NK, AN, KKa, SC, AH, EA_A, and SP performed the statistical analysis and drafted the manuscript. All authors contributed to manuscript revision, read, and approved the submitted version.

## Conflict of Interest

The authors declare that the research was conducted in the absence of any commercial or financial relationships that could be construed as a potential conflict of interest.

## Publisher’s Note

All claims expressed in this article are solely those of the authors and do not necessarily represent those of their affiliated organizations, or those of the publisher, the editors and the reviewers. Any product that may be evaluated in this article, or claim that may be made by its manufacturer, is not guaranteed or endorsed by the publisher.
