# Supplementary material for: Multi-Biofunctional Properties of Phytofabricated Selenium Nanoparticles From Carica papaya Fruit Extract: Antioxidant, Antimicrobial, Antimycotoxin, Anticancer, and Biocompatibility
Source: Front Microbiol. 2022 Feb 17;12:769891. doi: 10.3389/fmicb.2021.769891 (PMC8892101; doi:10.3389/fmicb.2021.769891)
Supplement: Supplementary file 1 [file Data_Sheet_1.docx]

**Supplementary data**

**Supplementary Materials and Methods**

**1. Chemicals and reagents**

Syringe filters of 0.22 µm, Dulbecco's modified Eagle's medium (DMEM), ochratoxin A (OTA) of 98% unadulterated grade of HPLC (CAS no: 303-47-9), Caspase-3 assay kit, Dulbecco's phosphate-buffered saline (DPBS), sodium selenite (CAS no: 10102-18-8), trypsin, and Whatman no.1 filter papers were procured from Sigma-Aldrich, Andhra Pradesh, India. Mueller-Hinton broth and agar, streptomycin (CAS no:3810-74-0), cell lysis buffer, rhodamine 123 (CAS no: 62669-70-9), 4′,6-diamidino-2-phenylindole (DAPI) (CAS no: 28718-90-3), peptone (CAS no: 100209-45-8), Czapek dox medium, fetal bovine serum (FBS), dichloro-dihydro-fluorescein diacetate (DCFH-DA) (CAS no: 4091-99-0), (3-(4,5-dimethylthiazol-2-yl)-2,5-diphenyltetrazolium bromide (MTT) (CAS no: 57360-69-7), and penicillin (CAS no: 69-57-8 ) were procured from HiMedia, Andhra Pradesh, India. Cell culture plasticware was obtained from Eppendorf, Andhra Pradesh, India, and other chemicals used in the study from Merck Millipore Corporation, Andhra Pradesh, India. The rapid and precision immunoaffinity clean-up column of OTA was from VICAM, Water Business, USA.

**2.** **Quantification of total phenolics**

Briefly, one mL of *C. papaya* fruitextract was diluted three times with distilled water and mixed with 0.5 mL of Folin-Ciocalteau reagent and 1 mL of Na₂CO₃ (7.5%). The reaction mixture was nurtured for 45 min at room temperature in the dim and a microplate reader was used to note the transmission intensity of the reaction mixture at 765 nm. Gallic acid was measured as a standard phenolic in this work, and the results were stated as mg of gallic acid equivalents per gram of *C. papaya* fruit extract (mg GAE/g).

**3.** **Quantification of total flavonoids**

Briefly, one mL of *C. papaya* fruitextract was amalgamated with 140 μL of 5% NaNO₂ and nurtured at room temperature aimed at 15 min. Later, the combination was merged with 1 mL of 1M NaOH, 0.30 mL of 10% AlCl₃, and 2.5 mL of purified water. Subsequently, experimental mixture was nurtured for 15 min at room temperature and transmission intensity was noted at 415 nm using the microplate reader.The reference flavonoid used in the experiment was quercetin, and the results were stated as mg of quercetin equivalents per gram of *C. papaya* fruit extract (mg QUE/g).

**4.** **Phytofabrication and characterization of SeNPs**

The transmission intensityof SeNPs was measured in the range of 200 to 800 nm using an Agilent-Cary 60 UV–visible spectrophotometer (USA). The role of *C. papaya* fruit extract in the phytofabrication of SeNPs was assessed using FTIR spectroscopy, which was done based on functional groups of molecules and carried out using a Thermo Nicolet 6700 FTIR spectrophotometer (USA) (Gunti et al., 2019).Zetasizer, Nano S, Malvern Instrument (United Kingdom) was used to evaluate the hydrodynamic size distribution and charge of SeNPs. The phase formation of SeNPs was characterized in Debye-Scherrer mode using a PANalytical apparatus coupled with a Cu anode as an X-ray source (Gunti et al., 2019). The morphological and elemental examination of SeNPs were done using the scanning electron microscope (SEM) and energy-dispersive X-ray (EDX), respectively, the assays were carried out using equipment Icon Analytical, Quanta 250, FEI (India) (Gunti et al., 2019).To precisely analyze the size and shape of SeNPs, a high-resolution transmission electron microscope (HR-TEM), JEM-2100, JEOL Ltd., (Japan) was utilized (Gunti et al., 2019).

**5. DPPH assay**

Briefly, varying amounts of SeNPs (up to 80 µg/mL) were combined to 0.5 mL of methanolic DPPH radical solution (0.1 mM) and 1 mL of 0.1 M acetate buffer, and the total volume was brought up to 3 mL with methanol. The solution was incubated at room temperature for 20 min under complete darkness before being evaluated for optical density (OD) at 515 nm. The standard antioxidant in the study was ascorbic acid. The reaction mixture alone was utilized as a control. The antioxidant potential of SeNPs was expressed in EC_50_ value, which is the amount of SeNPs necessary to reduce the optical density of DPPH radical solution by about half.

The following formula was used to compute the percentage of DPPH radical scavenging:

$$DPPH radical scavenging activity (\%)=\frac{\left( S_{c}-S_{t} \right)}{S_{c}}\times100$$

Where the terms ‘S_t_’ and ‘S_c_’ in the equation refer to the test and control’s absorbances, respectively.

**6.** **ABTS assay**

Concisely, ABTS radical solution of 7 mM was prepared in potassium persulfate of 2.4 mM and optical density was set at 0.7. A quantity of 3 mL of ABTS radical solution was added to various quantities of SeNPs (up to 80 µg/mL) and incubated for 8 min at room temperature under complete darkness. Following, a microplate reader was used to measure the optical density at 730 nm.Ascorbic acid was used as a standard antioxidant in the study. The reaction mixture alone was utilized as a control.The EC_50_ value of SeNPs was calculated as the amount of SeNPs required to reduce the optical density of the ABTS radical solution by 50%.

The percentage of ABTS radical scavenging activity was calculated using the following formula:

$$ABTS radical scavenging activity (\%)=\frac{\left( S_{c}-S_{t} \right)}{S_{c}}\times100$$

Where the terms ‘S_t_’ and ‘S_c_’ in the equation refer to the test and control’s absorbances, respectively.

**7. Antibacterial activity**

Bacteria were cultured for 24 hrs at 37 °C in Mueller-Hinton broth medium, and OD was measured at 600 nm using a multiplate reader. With sterile phosphate-buffered saline of pH 7.4 (PBS), the OD of bacterial culture was adjusted to 0.5 McFarland standard and employed in the micro-well dilution technique (Zapata & Ramirez-Arcos, 2015). Briefly, SeNPs at various concentrations (up to 65 µg/mL)and overnight bacterial culture of 0.5 McFarland (10 µL) were added to wells of a 96-well plate, and the final volume was adjusted to 0.1 mL with Muller Hinton broth and cultured at 37 °C for 24 hrs. Subsequently, OD was measured at 600 nm using a microplate reader. The MIC of SeNPs is defined as the concentration at which bacterial growth is inhibited. Furthermore, 25 µL was collected and spread out on Muller Hinton agar, which was then incubated at 37 °C for 24 hrs. The MBC of SeNPs is defined as the concentration at which bacterial growth was could not reoccur. Tetracycline was referred to as a standard antibacterial drug.

**8. Antifungal activity**

Fungi were cultivated for 7 days at 28 °C on Sabouraud dextrose agar plates. Soft scraping was used to extract fungal spores in sterile peptone broth with 0.01% Tween 80. A hemocytometer was used to determine the spore count, which was corrected to 1 × 10^6^ per mL and employed in the antifungal activity (Kalagatur et al., 2020). SeNPs of various concentrations (up to 45 µg/mL)and 10 µL of three-day-old fungal spores (1 × 10^6^ per mL) were added to the wells of a 96-well plate, and the total volume was fixed to 0.1 mL with Czapek dox broth and incubated for three days at 28 °C. The MIC was defined as the concentration of SeNPs at which no detectable fungal growth was found. Furthermore, 25 µL of the sample was taken and spread out on Czapek dox agar, which was then incubated at 28 °C for three days. The MFC of SeNPs is defined as the concentration at which fungal development is not grown again. Nystatin was a standard antifungal drug.

**9. Quantification of fungal growth and OTA**

The fungal mycelia (biomass) were collected in pre-weighed Whatman no.1 filter paper after being properly washed twice with distilled water. The mycelia were dried at 60 °C in a hot-air oven with pre-weighed Whatman no.1 filter paper, and the fungal biomass was precisely weighed.

HPLC was used to quantify the OTA. Briefly, the culture filtrate was mixed evenly with acetonitrile (1:1, v/v) and blended under 140 rpm shaking for 90 min.Following that, the clear supernatant was collected at 8,000 rpm and filtered using Whatman no.1 filter paper. A volume of 15 mL of filtrate was passed over an OTA selective immunoaffinity column at a passage rate of 2.5 mL per min in an instantaneous. The immunoaffinity clean-up of OTA was done according to the manufacturer's instructions. The obtained eluate was utilized to measure OTA by HPLC as per the method of Kalagatur et al., (2020).The standard calibration curve of OTA was constructed for the measurement of OTA in test samples. The regression coefficient was used to determine the precision of the standard calibration curve, which was determined to be 0.981 on a scale of one. The limit of detection (LOD), limit of quantification (LOQ), and recovery of the technique were found to be 20.21 ± 2.42 ng/mL, 92.81 ± 4.92 ng/mL, and 98.37 ± 2.81%, respectively.

**10. Cell viability and micro-morphology assessment**

Approximately, 1.8 × 10^3^ cells were plated in a 96-well cell culture plate, allowed to adhere for 8 hrs, and then exposed to different dosages of SeNPs(up to 120 µg/mL) for 24 hrs.The cells treated with cisplatin (up to 10 µg/mL) for 24 hrs was referred as positive control.An inverted bright-field microscope was used to obtain subsequent microscopic photographs of cells. After that, a culture medium was removed and 10 µL of MTT reagent (5 mg/mL in DPBS) was added and incubated for four hrs.To this, 90 µL of DMSO was added to dissolve formazan crystals and incubated for three hrs. Following, absorbance was measured at 570 nm in the multiplate reader. The viability of SeNPs-exposed cells was calculated as a percentage of the viability of control cells. Following, IC_50_ (dose of SeNPs required to inhibit 50% cell viability) and IC_90_ (dose of SeNPs required to inhibit 90% cell viability) values of SeNPs and cisplatin against cancer and normal cells were calculated. The cell viability was calculated using the formula,

$$Cell viability (\%)=\frac{A_{t}}{A_{c}}\times100$$

Where, terms ‘A_t_’ and ‘A_c_’ in the equation refer to the absorbance of the test and control samples, respectively.

**11. LDH analysis**

The assay was achieved following the methodology of Kalagatur et al., (2021), and instructions of the LDH assay kit (Sigma-Aldrich). Briefly, cells were plated at a density of 5.5 × 10^4^ cells per well in a 24-well cell culture plate, allowed to settle for 8 hrs, and then administered for 24 hrs with various doses of SeNPs (up to 120 µg/mL) in 250 µL of DMEM devoid of FBS. Next, supernatant from the cell culture was then collected at 3,500 rpm for 3 min at 4 ºC, 100 µL was used to assess LDH release, and the results were expressed as a percentage of LDH release in comparison to control cells.

**12. MMP analysis**

Briefly, Cells at a density of 5.5 × 10^4^ cells/well were introduced in a 24-well cell culture plate and allowed to adhere for 8 hrs before being administered with various dosages of SeNPs (up to 120 µg/mL) for 24 hrs. Cells that were not treated with SeNPs were used as controls. Following, the cells were stained with 5 µM of MMP specific dye rhodamine 123 for 20 min, washed repeatedly with DPBS for thrice, and OD was measured in a multiplate reader at an excitation of 485 nm and an emission of 535 nm. MMP levels in SeNPs-exposed cells were expressed as a percentage of control cells.

**13. Caspase-3 analysis**

In a 24-well cell culture plate, cells at a population of 5.5 × 10^4^ cells/well were seeded and left to adhere for eight hrs before being exposed with varying dosages of SeNPs (up to 120 µg/mL) for 24 hrs.Following that, cells were collected by trypsinization and subjected to wash with DPBS for twice before being lysed in ice-cold lysis buffer and the lysate was obtained at 4 ºC for 5 min at 10,000 rpm. Using a multiplate reader at 405 nm, the chromophore ρ-nitroanilide (p-NA) released from the tagged substrate, acetyl-Asp-Glu-Val-Asp ρ-nitroanilide, was quantified in the lysate. Caspase-3 activity in SeNPs-treated cells was expressed as a percentage of control cells. Control cells were those that had not been exposed to SeNPs.

**14. *In-vivo* toxicological assessment in *Danio rerio***

The *D. rerio* is chosen because it is an ideal vertebrate model organism and transparency of *D. rerio* embryos consents to the constant observation of developmental changes as they progress through organogenesis (Hill et al., 2005). Disintegration or coagulation and the absence of a heartbeat of embryos during development are referred to as dead embryos. Deformation during embryogenesis is defined as non-tail detachment, absence of somite development, translucent eyes, pericardial edema, yolk sac edema, hyperemia, and spinal curvature.

Briefly, wild-type adult *D. rerio* were purchased from a local aquarium market in Coimbatore, Tamil Nadu, and maintenance and acclimatization of *D. rerio* were done as per recommendations of OECD, (2013).The average body weight of female and male fish was 0.66 ± 0.01 g and 0.48 ± 0.08 g, respectively. The fish breeding (2 males:1 female), eggs collection, and embryotoxicity test was carried out according to the OECD 236 guidelines (OECD, 2013).The embryos (n = 50 per group) were individually exposed with different concentrations of SeNPs (10, 25, 50, 75, and 100 µg/mL in embryo medium) in 24-well plates (2 mL/well) from 4 to 96 hrs post-fertilization (hpf). The experiment consists of five groups including control and four SeNPs treatment groups (25, 50, 75, and 100 µg/mL). The embryos not exposed to SeNPs were control. Following, mortality and developmental abnormalities of embryos were observed under an inverted microscope at 4x magnification (EVOS FLC, Life technologies, USA) at different time intervals viz., 24, 48, 72, and 96 hpf of SeNPs exposure.

**Supplementary Tables**

**Supplementary Table1:** Antimicrobial activity of SeNPs determined by micro-well dilution assay

|  | Antimicrobial concentration of SeNPs | |
| --- | --- | --- |
|  | MIC (µg/mL) | MBC/MFC (µg/mL) |
| Bacteria |  |  |
| *E. faecalis*– MTCC 439 | 26.11 ± 2.04^a^ | 43.32 ± 4.21^b^ |
| *E. coli* – MTCC 41 | 31.57 ± 1.89^a^ | 58.52 ± 3.67^b^ |
| *L. monocytogenes* – MTCC 657 | 22.56 ± 3.11^a^ | 38.56 ± 2.59^b^ |
| *S. aureus* – MTCC 96 | 24.82 ± 4.83^a^ | 39.68 ± 3.18^b^ |
| *S. aureus* – ATCC 13565 | 26.39 ± 3.95^a^ | 41.17 ± 3.76^b^ |
| *S. aureus* – ATCC 14458 | 26.70 ± 2.81^a^ | 40.09 ± 2.68^b^ |
| *P. aeruginosa–*MTCC 741 | 33.47 ± 3.09^a^ | 61.14 ± 3.94^b^ |
| Fungi |  |  |
| *P. verrucosum*– ITCC 2986 | 16.84 ± 2.04^a^ | 30.58 ± 3.91^b^ |
| *A. ochraceus* – ITCC 2454 | 16.22 ± 1.81^a^ | 28.41 ± 4.07^b^ |
| *A. oryzae* – MTCC 634 | 18.55 ± 2.03^a^ | 29.35 ± 2.48^b^ |
| *F. anthophilum*– MTCC 10129 | 27.88 ± 3.56^a^ | 41.90 ± 5.22^b^ |
| *A. brasiliensis* – MTCC 1344 | 20.30 ± 1.81^a^ | 32.68 ± 3.58^b^ |
| *A. flavus*– MTCC 1883 | 21.16 ± 2.39^a^ | 33.74 ± 4.14^b^ |
| *R. stolonifer* – MTCC 4886 | 22.81 ± 1.37^a^ | 35.59 ± 2.55^b^ |

The rows with different alphabets within the respective microorganisms were significant.

**Supplementary Table 2:**IC_50_ and IC_90_ values of cisplatin and SeNPs in cancer cells (RAW 264.7, Caco-2, MCF-7, and IMR-32 cells) and normal cells (Vero cells) determined by MTT assay

| Cells | Concentration of cisplatin (µg/mL) | |  | Concertation of SeNPs (µg/mL) | |
| --- | --- | --- | --- | --- | --- |
|  | IC_50_ | IC_90_ |  | IC_50_ | IC_90_ |
| RAW 264.7 | 3.03 ± 0.08^a^ | 5.30 ± 0.27^b^ |  | 38.76 ± 2.68^a^ | 73.85 ± 4.00^b^ |
| Caco-2 | 3.23 ± 0.11^a^ | 5.56 ± 0.34^b^ |  | 42.85 ± 2.02^a^ | 79.55 ± 3.97^b^ |
| MCF-7 | 3.19 ± 0.13^a^ | 5.55 ± 0.24^b^ |  | 41.22 ± 3.21^a^ | 77.26 ± 4.14^b^ |
| IMR-32 | 2.87 ± 0.06^a^ | 5.05 ± 0.19^b^ |  | 35.98 ± 2.89^a^ | 71.38 ± 3.71^b^ |
| Vero | 5.72 ± 0.29^a^ | 9.62 ± 0.53^b^ |  | 58.55 ± 3.08^a^ | 109.68 ± 5.82^b^ |

The rows with different alphabets within the respective cells were significant.

**Supplementary Figures**


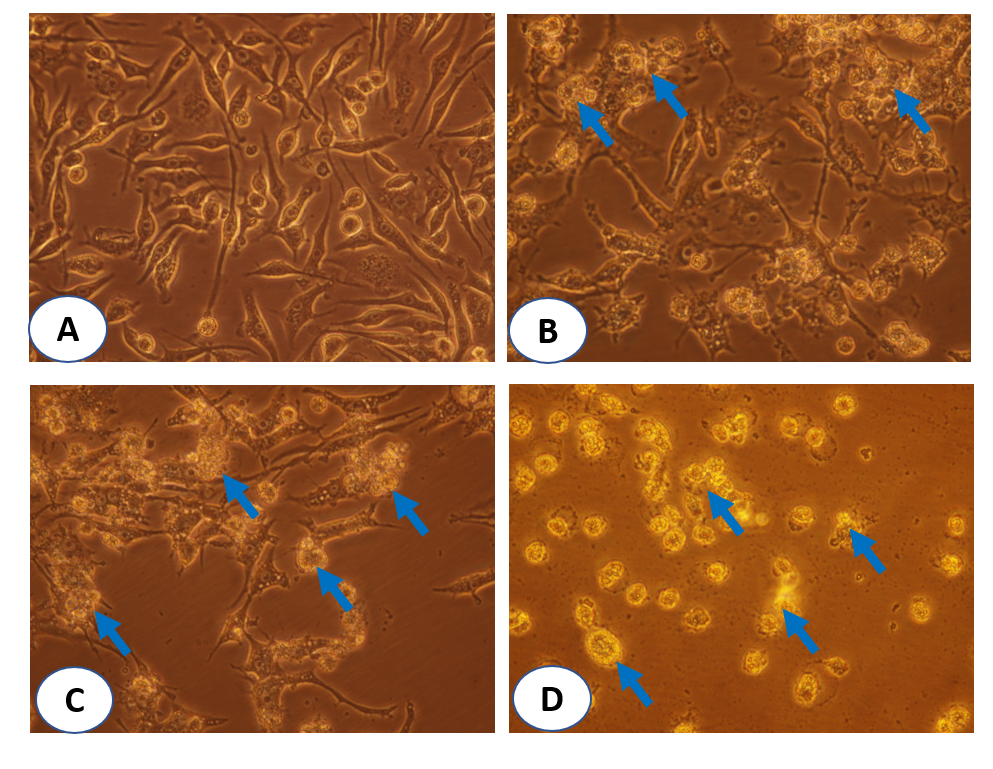


**Supplementary Figure 1:**Effect of selenium nanoparticles (SeNPs) and cisplatin on micro-morphology of RAW 264.7 cells observed by bright-field microscope. (**A)** Control cells. (**B)** Cells treated with IC50 value of cisplatin (3.03 ± 0.08 µg/mL). **(C)** Cells treated with IC50 value of SeNPs (38.76± 2.68 µg/mL). **(D)** Cells treated with IC90 value of SeNPs (73.85± 4.00 µg/mL). The arrows indicate the cellular membrane damage and cellular debris. The images were shown at a magnification of 400x.


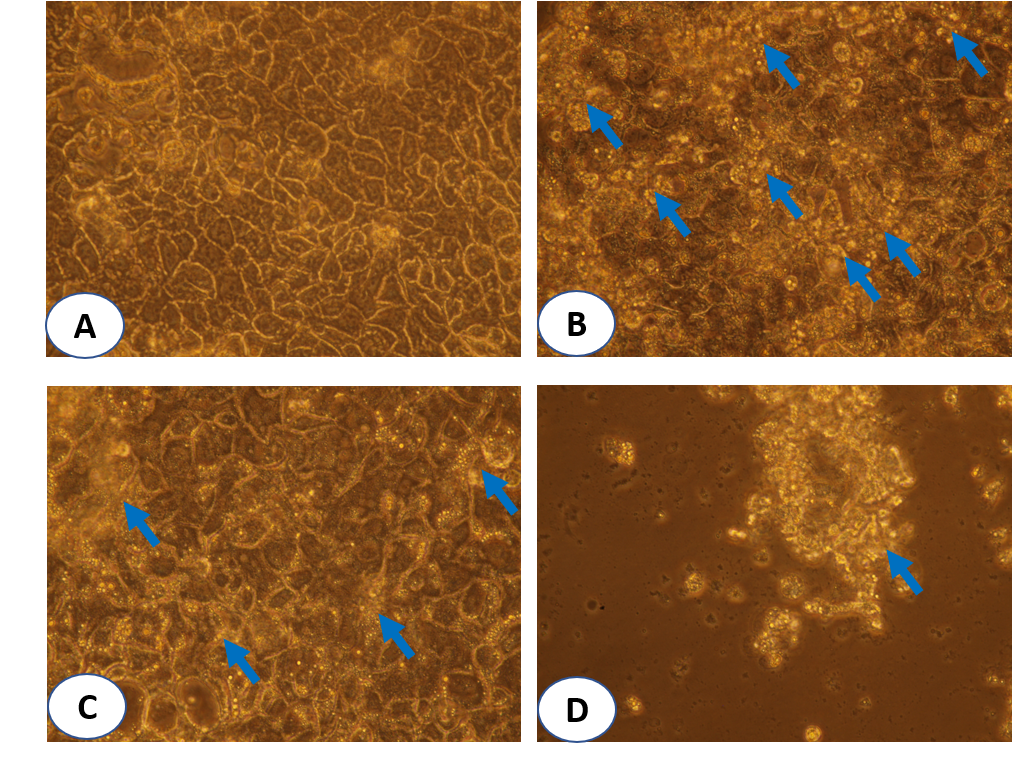


**Supplementary Figure 2:** Effect of selenium nanoparticles (SeNPs) and cisplatin on micro-morphology of Caco-2cells observed by bright-field microscope. (**A)** Control cells. (**B)** Cells treated with IC50 value of cisplatin (3.23 ± 0.11 µg/mL). **(C)** Cells treated with IC50 value of SeNPs (42.85± 2.02 µg/mL). **(D)** Cells treated with IC90 value of SeNPs (79.55± 3.97 µg/mL). The arrows indicate the cellular membrane damage and cellular debris. The images were shown at a magnification of 400x.


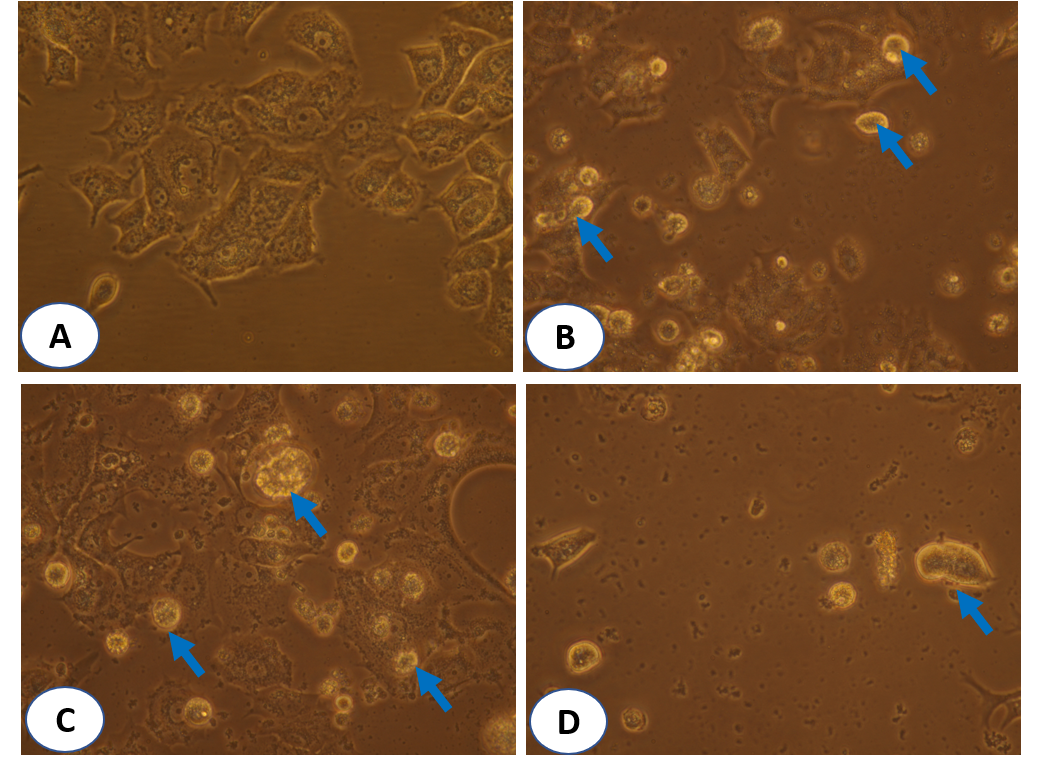


**Supplementary Figure 3:** Effect of selenium nanoparticles (SeNPs) and cisplatin on micro-morphology of MCF-7 cells observed by bright-field microscope. (**A)** Control cells. (**B)** Cells treated with IC50 value of cisplatin (3.19 ± 0.13 µg/mL). **(C)** Cells treated with IC50 value of SeNPs (41.22± 3.21 µg/mL).**(D)** Cells treated with IC90 value of SeNPs (77.26± 4.14 µg/mL). The arrows indicate the cellular membrane damage and cellular debris. The images were shown at a magnification of 400x.


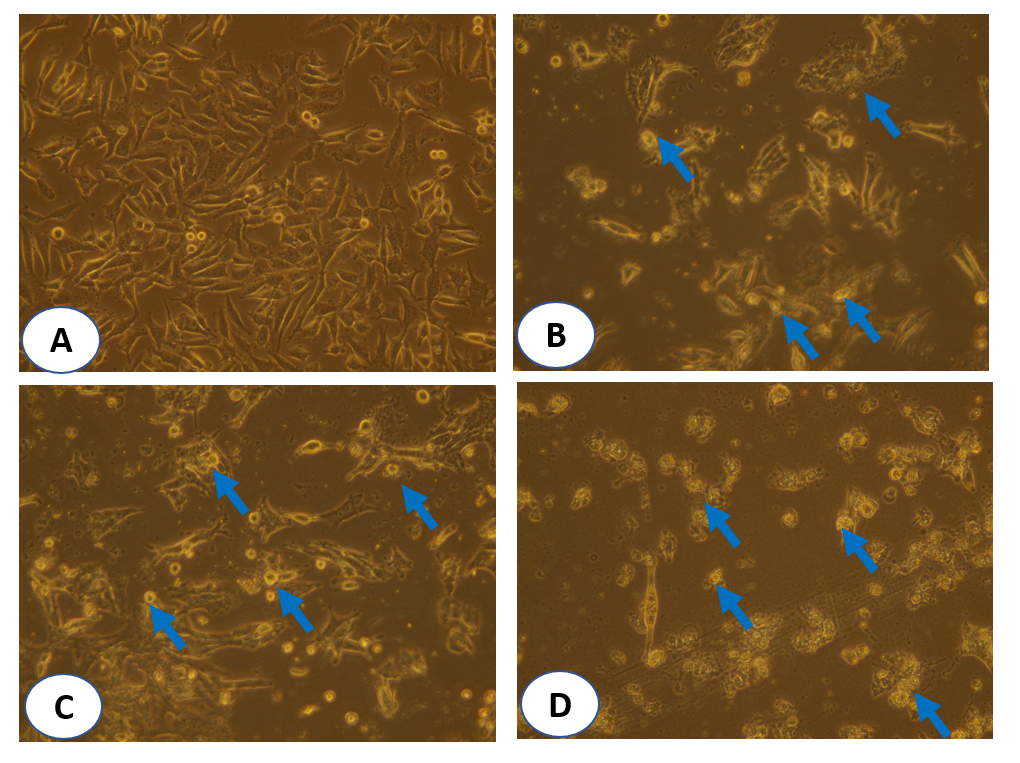


**Supplementary Figure 4:** Effect of selenium nanoparticles (SeNPs) and cisplatin on micro-morphology of IMR-32cells observed by bright-field microscope. (**A)** Control cells. (**B)** Cells treated with IC50 value of cisplatin (2.87 ± 0.06 µg/mL). **(C)** Cells treated with IC50 value of SeNPs (35.98± 2.89 µg/mL). **(D)** Cells treated with IC90 value of SeNPs (71.38± 3.71 µg/mL). The arrows indicate the cellular membrane damage and cellular debris. The images were shown at a magnification of 400x.


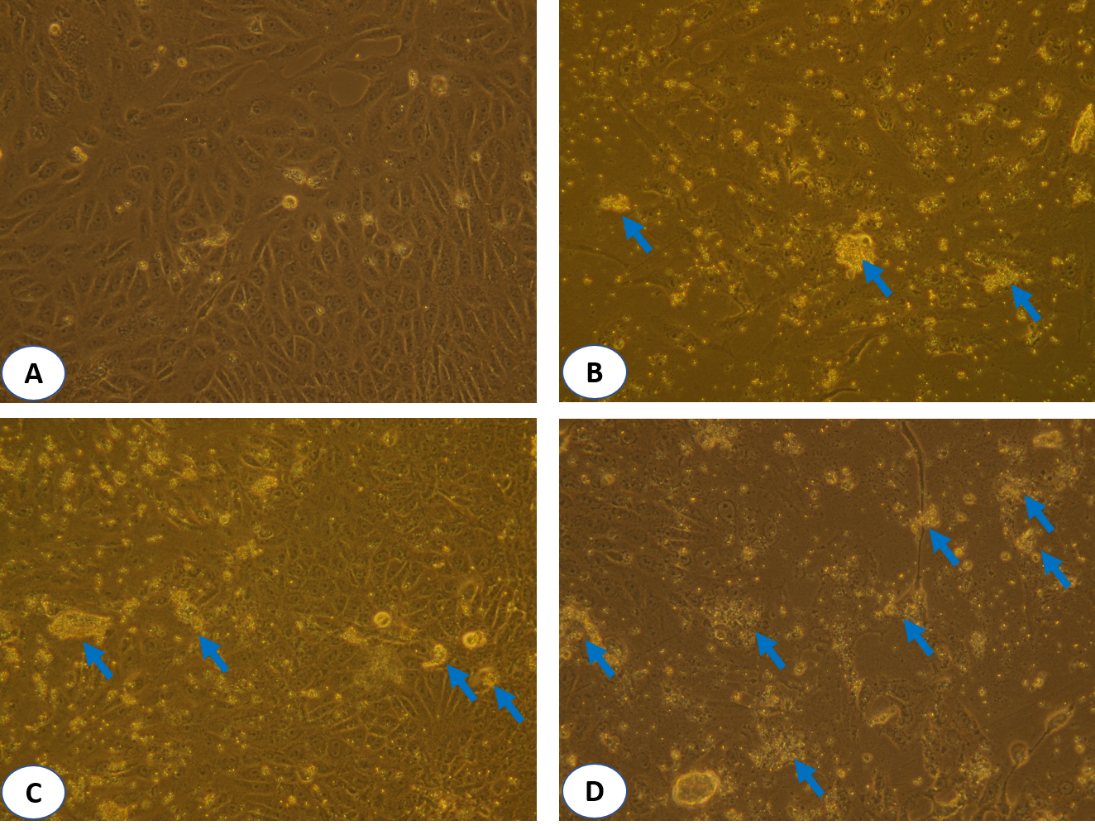


**Supplementary Figure 5:** Effect of selenium nanoparticles (SeNPs) and cisplatin on micro-morphology of Vero cells observed by bright-field microscope. (**A)** Control cells. (**B)** Cells treated with IC50 value of cisplatin (5.72 ± 0.29 µg/mL). **(C)** Cells treated with IC50 value of SeNPs (58.55 ± 3.08 µg/mL). **(D)** Cells treated with IC90 value of SeNPs (109.68 ± 5.82 µg/mL). The arrows indicate the cellular membrane damage and cellular debris. The images were shown at a magnification of 400x.
